# Utilizing weakly donor–acceptor ternary π-conjugated architecture to achieve single-component white luminescence and stimulus-responsive room-temperature phosphorescence†

**DOI:** 10.1039/d4sc02525c

**Published:** 2024-07-05

**Authors:** Wenbin Huang, Yuxin Zhu, Xinwei Xie, Guanqun Tang, Kang Zhou, Lijuan Song, Zikai He

**Affiliations:** a School of Science, Harbin Institute of Technology Shenzhen Shenzhen Guangdong 518055 China; b School of Civil and Environmental Engineering, Harbin Institute of Technology Shenzhen Shenzhen Guangdong 518055 China; c Hoffman Institute of Advanced Materials, Shenzhen Polytechnic University Shenzhen Guangdong 518055 China

## Abstract

Purely organic room-temperature phosphorescence (RTP) has garnered substantial attention for its delayed emission, environmental sensitivity, and potential diverse applications. However, the quest for high-performance RTP materials has always been a challenge. In this study, we introduce novel weakly donor–acceptor (D–A) ternary π-conjugated architecture to construct an efficient RTP system. The strategy utilizes synergistic effects of the analogous El-Sayed rule, halogen-free heavy-atom effect, reduction of the singlet–triplet energy gap, and manipulation of flexible molecular conformation. A remarkable enhancement in the phosphorescence-to-fluorescence ratio was achieved, elevating from 0.4 in carbazole to 35.2 in DBTDBTCZ. Furthermore, the RTP system demonstrates single-component white luminescence, yielding warm and cool white colors. Intriguingly, we unveil the novel position-dependent heavy-atom effects, discerningly promoting intersystem crossing or phosphorescence decay. Benefiting from efficient RTP, multifunctional applications of real-time humidity monitoring, oxygen sensing, anti-counterfeiting labeling, and white lighting are demonstrated.

## Abstract 2

Weakly D–A ternary π-conjugated skeleton is proposed to improve room temperature phosphorescence through synergistic strategies, and achieves high phosphorescence proportion and stimulus-responsive emission, and single-component white luminescence.

## Graphical abstract

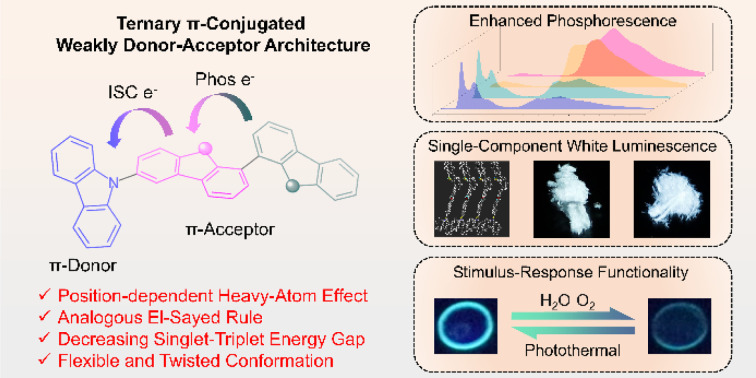

## Introduction

Purely organic room-temperature phosphorescence (RTP) has given rise to a new generation of organic optoelectronic materials and biomedical agents.^1–4^ Versatile applications such as X-ray organic scintillation, efficient electroluminescence, free-background bioimaging, and information encryption have been demonstrated.^5–8^ However, limited by spin-forbidden intersystem crossing (ISC) and the high sensitivity of triplet excitons, efficient RTP systems are still difficult to realize.^9,10^ Strategies such as the heavy-atom effect,^11–14^ El-Sayed rule,^15–18^ reducing singlet–triplet energy gap,^19,20^ restricting intramolecular^21,22^ and intermolecular motions,^23,24^*etc.* have been employed to overcome the weak ISC process and eliminate the quenching factors. It is worth noting that rational molecular structural engineering always supports the effectiveness of these strategies, as well as provides additional functionalities such as single-component white luminescence^25,26^ and stimulus-responsive behavior.^27–29^ Therefore, adopting a comprehensive molecular structural design strategy to improve RTP performance, and exploring novel applications such as lifetime-dependent afterglow, chemosensing, and white illumination is the focus of developing functional RTP materials.

**Scheme 1:**
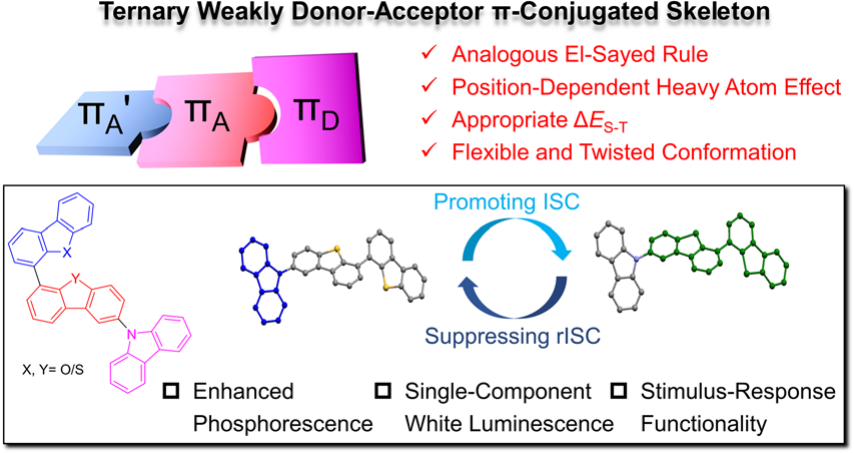
The strategies in constructing a weakly donor–acceptor ternary π-conjugated skeleton and diagrammatic molecular structural engineering.

In this study, we developed a series of weakly donor–acceptor (D–A) ternary π-conjugated RTP molecules with the aim of rational molecular structural engineering. Strategies of the analogous El-Sayed rule, halogen-free heavy-atom effect, reduced singlet–triplet energy gap (Δ*E*_ST_), and the manipulation of flexible molecular conformation were applied synergistically to boost the RTP performance (Scheme 1). Consequently, the weakly D–A ternary π-conjugated skeleton exhibited a remarkable increase in the phosphorescence-to-fluorescence ratio. Notably, a position-dependent heavy-atom effect favoured intersystem crossing when close to the excited region but accelerated phosphorescence decay when close to the emitting region. The finely tuned phosphorescence and the face-to-face π–π stacking from a flexible molecular conformation were responsible for the single-component cold and warm white luminescence. Finally, doping phosphors into a treated PVA film offers an excellent environment matrix for facilitating bright RTP emission and resulting stimulus-response behaviour towards moisture and oxygen.

## Materials and methods

### Synthesis of ternary π-conjugated compounds

Detailed synthetic routes are presented in the ESI (Scheme S1, Charts S1–S18†). The single crystals are obtained by dissolving the compounds in tetrahydrofuran and subsequently allowing the solvent to evaporate gradually.

### Materials

The samples were repeatedly purified by silica-gel column chromatography, gel exclusion chromatography, and recrystallization before being submitted to photophysical property measurement (Chart S19, ESI†). 2-Methyltetrahydrofuran (2-MeTHF) with high-performance liquid chromatography purity was further distilled for photophysical solution preparation.

### Preparation of doped PVA films

Poly(vinyl alcohol) (PVA) was purchased from J&K Scientific (88% hydrolysed) and used without further purification. Doped films were prepared: 100 mg PVA was dissolved in 5 mL deionized water at 373 K for 10 min to afford a homogeneous PVA aqueous solution. The organic molecules (1 mg/50 mL in THF) were added to prepared PVA solutions. The obtained solutions were then ultrasonicated for 30 min. The doped PVA films were produced with the drop-casting approach on quartz glass and were allowed to naturally dry at room temperature. As-prepared films were thermally annealed at 393 K for 30 minutes and then photo-activated by 254 nm ultraviolet light at room temperature for 10 minutes to obtain treated PVA film. The as-prepared films and treated films were selected for photophysical characterization.

## Results and discussion

### Molecular design and synthesis

According to the energy gap law, small Δ*E*_ST_ is beneficial for forward and reverse ISC, which is tuned by separating the highest occupied molecular orbital (HOMO) and the lowest unoccupied molecular orbital (LUMO) in D–A systems. The significant electronic push–pull effect can dramatically separate the HOMO and LUMO spatial distribution, resulting in a nearly isoenergic lowest singlet excited state (S_1_) and triplet state (T_*n*_) (Δ*E*_ST_ ∼ 0 eV). The strategy has been widely utilized in achieving thermally activated delayed fluorescence.^30^ However, it is not suitable for designing an efficient RTP system, as RTP phosphors should emit from the lowest triplet excited state (T_1_), which stems from T_*n*_ through internal conversion, competing with the reverse ISC process. Thus, considerable energy gap between T_*n*_ and S_1_ is needed to block the reverse ISC process. The weak D–A molecular structure can solve the problem as the extent of intramolecular charge-transfer (CT) character becomes insignificant to open the degenerated S_1_ and T_*n*_. Also, a weak D–A structure endows more locally excited (LE) character of excited states to enlarge the energy gap of Δ*E*_T_1_–T_*n*__. Therefore, we propose that the weak D–A scaffold could realize efficient RTP, where a reduced Δ*E*_ST_ and the inclusion of LE character will accelerate the ISC process *via* a second-order vibronic coupling mechanism,^31^ and the hybridized orbit configurations of CT and LE characters can separate energy levels between T_1_ and T_*n*_. Moreover, strategies such as the classic El-Sayed rule^16,32,33^ and heavy-atom effect^34,35^ are also powerful in boosting the efficiency of RTP systems. They are further developed in these weakly D–A and twisted ternary π-conjugated skeletons. Similar to the p_*x*_ → p_*y*_ transition, the analogous El-Sayed rule utilizes the circular electron motions between the twisted π-segments to fulfil the angular momentum conservation and avoids the need for carbonyl groups and the photodegradation drawback. Conjugated sulphur atoms in π-segments not only exert the position-dependent halogen-free heavy-atom effect but also exclude the photobleaching and thermal decomposition of carbon–halogen bonds.^36–39^

**Scheme 2:**
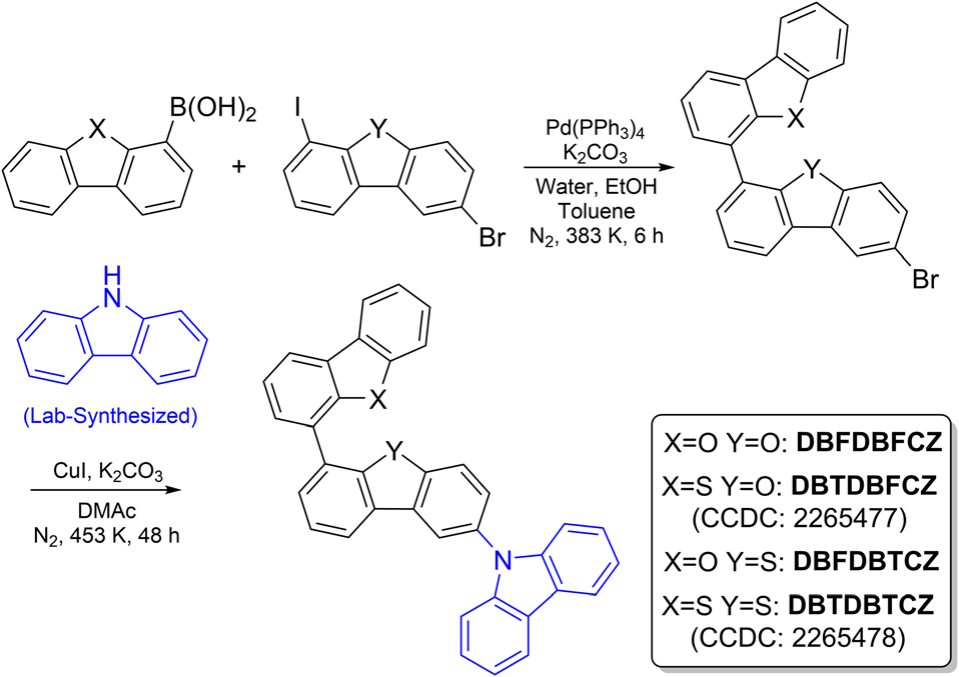
Synthetic routes of investigated ternary π-conjugated molecules.

Collectively, weakly D–A molecules with different π-fragments were designed and synthesized following the procedures shown in Scheme 2. Classic π-segments were selected, including carbazole (CZ), dibenzofuran (DBF), and dibenzothiophene (DBT). Notably, CZ was synthesized by a one-step coupling reaction of 2-bromodiphenylamine to avoid isomer-induced fake phosphorescence in commercial products.^40,41^ DBF and DBT are chosen as weak π-acceptors and interconnected in a rotary pattern through the Suzuki coupling. Tunable (n,π*) and (π,π*) characters are introduced by virtue of through-bond conjugation and lone-pair electrons. Then, CZ is attached as the π-donor to form D–A–A′ skeletons by the Ullmann coupling. The four obtained compounds were repeatedly purified by silica-gel chromatography and preparative gel-permeation chromatography, and recrystallized before being submitted for photophysical property measurement. For comparison, binary π-conjugated molecules of DBFDBF, DBFDBT, and DBTDBT were also prepared (Scheme S1, ESI†).

### Photophysical properties

**Fig. 1:**
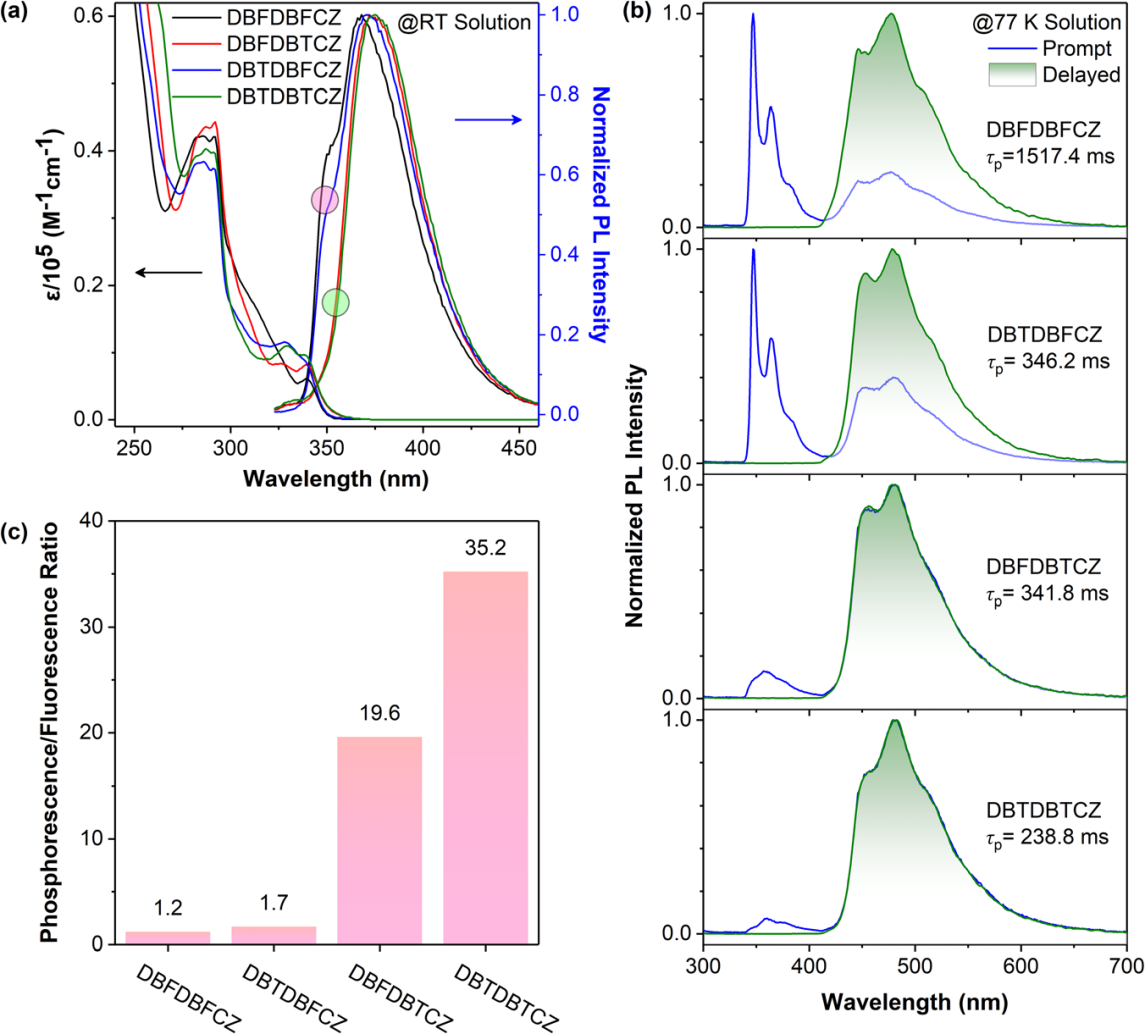
(a) UV-visible absorption spectra and normalized PL spectra at room temperature, (b) normalized PL prompt and delayed (1 ms) spectra at 77 K, (c) the integral area ratio of phosphorescence to fluorescence at 77 K of DBFDBFCZ, DBTDBFCZ, DBFDBTCZ, and DBTDBTCZ in 2-MeTHF (10^−5^ M).

Photophysical properties of unimolecular compounds were first investigated in 2-MeTHF solutions. Their overall absorption profiles are similar, with an absorption band at 320–350 nm corresponding to the S_1_ (Fig. 1a). The absorption similarity indicates the same excitation processes of the S_1_ (Fig. S4, ESI†), corresponding to the excitation of π-electrons in the carbazole segment to the π*-orbit in DBF(DBT) binary skeletons. The solvent polarity has no influence on absorption but causes a slight redshift in fluorescence (Fig. S8, ESI†). The calculated HOMOs, LUMOs, energy gaps, and natural transition orbits illustrate the fact of the weakly D–A characteristic of these ground-state lumiphores (Fig. S1 and S2, ESI†), where the CZ serves as the electron donor and DBF/DBT binary skeletons serve as the electron acceptors. From the similar onset wavelengths of the absorption spectra of π-segments, binary and ternary skeletons, the D–A extent of ternary π-conjugated molecules is relatively weak. The overall conjugation is poor, considering the low absorption coefficients and the small oscillator strengths (Fig. 1a and S2, ESI†).

In contrast, notable differences in photoluminescence (PL) spectra were found. Fig. 1a and b illustrate that unimolecular fluorescence with distinct vibration characteristics was observed with lifetimes in the nanosecond range (Table S3, ESI†). DBFDBFCZ and DBTDBFCZ (red cycle), DBFDBTCZ and DBTDBTCZ (green cycle) showed different emission profiles, respectively. The groups directly linked to CZ play critical roles. When the temperature was decreased to 77 K, a prominent and vibrational phosphorescence emission band emerged in the range of 440–600 nm with the lifetimes in the second range (Table S3, ESI†). Again, DBFDBFCZ and DBTDBFCZ showed similar emission profiles (Fig. 1b up) and phosphorescence-to-fluorescence ratios (Fig. 1c), different from the DBFDBTCZ and DBTDBTCZ (Fig. 1b bottom) counterparts, where CZ is directly attached to DBF and DBT segments, respectively.

To decipher the emission origin and the role of groups directly attached to CZ, we conducted a detailed comparison of the emission wavelengths, peak characteristics, and lifetimes of the isolated constructing π-fragments. As depicted in Fig. S5,† the fluorescence primarily originates from the CZ segment, whereas the phosphorescence arises from the binary π-conjugated skeletons of DBFDBF, DBFDBT, and DBTDBT, respectively. Interestingly, compared to the CZ, DBF, and DBT segments, both binary and ternary π-conjugated compounds exhibit a noticeable reduction in vibration characteristics, accompanied by an increased proportion of phosphorescence. This observation suggests the tendency for exciton delocalization and the evidence of hybridized ^1^(n,π*) orbit configurations. Moreover, an improved ISC process should be facilitated by the spin-allowed transitions between the ^1/3^(n,π*) and ^3/1^(π,π*) configurations. Together with twisted geometries of binary and ternary π-conjugated compounds, the systems could follow the analogous El-Sayed rule,^15^ where electrons go through circular motions among π-orbits from highly twisted aromatic segments. The experimental result proved that the effect of the analogous El-Sayed rule effectively promoted intersystem crossing, such as the higher phosphorescence-to-fluorescence ratio in DBTDBTCZ and DBTDBT compared with subcomponent DBT.

The incorporation of heteroatoms from O to S does not change the emission wavelength. Phosphorescence proportion or phosphorescence-to-fluorescence ratio can represent the phosphorescence performance in these systems. A significant increase in the phosphorescence proportion (the ratio of the phosphorescence emission area to the total PL spectra area) is found, indicating an enhancement in spin–orbit coupling (Fig. 1c). Consequently, the phosphorescence-to-fluorescence ratio increases from 1.2 in DBFDBFCZ to 35.2 in DBTDBTCZ, corresponding to the phosphorescence proportion from 53.7% to 97.2% (Fig. 1c and S6b, ESI†). A high phosphorescence-to-fluorescence ratio (or phosphorescence proportion) is crucial for the development of phosphorescent materials, as the purity of luminescence colour and high exciton utilization contribute to outstanding performance in luminescent devices.^42^ Furthermore, the lifetimes of phosphorescence and the quantum yields consistently display a decreasing trend from DBFDBFCZ (1517.4 ms, 20.6%) to DBTDBTCZ (238.8 ms, 2.3%). These findings suggest the evident heavy-atom effect of sulphur atoms (Table 1 and S3, ESI†). In particular, in contrast to DBTDBFCZ, DBFDBTCZ exhibits a significantly boosted phosphorescence proportion accompanied by a consistent phosphorescence lifetime. This phenomenon arises when the carbazole is located towards the heavy atom sulphur in different regions. The almost identical phosphorescence lifetime indicates that the binary π-conjugated skeleton DBFDBT contributes to the lowest triplet excited state (T_1_).

**Table 1:** Phosphorescence lifetime and Δ*E*_S_1_–T_1__ of DBF, DBFDBT, DBFDBFCZ, DBTDBFCZ, DBFDBTCZ, and DBTDBTCZ in 2-methyltetrahydrofuran at 77 K (10^−5^ M)

	DBF	DBFDBT	DBFDBFCZ	DBTDBFCZ	DBFDBTCZ	DBTDBTCZ
Phosphorescence lifetime (ms)	3075	443	1517	360	352	250
Δ*E*_S_1_–T_1__ (eV) (experimental)	1.15	0.87	0.66	0.65	0.65	0.67
Δ*E*_S_1_–T_1__ (eV) (calculated)	1.30	—	0.61	0.54	0.56	0.51

It is known that an excessively strong D–A structure results in a diminished singlet–triplet energy gap Δ*E*_ST_, leading to enhanced reverse ISC and thus decreased triplet exciton population.^43–45^ It is not suitable for achieving efficient RTP systems. The weakly D–A ternary π-conjugated structure is capable of separating the spatial distribution of HOMO and LUMO, but not heavily, which endows S_1_ with partial CT characteristics. For example, Δ*E*_ST_ is reduced from 1.15 eV for DBF to 0.87 eV for DBFDBT, and to 0.65 eV for DBFDBTCZ, consistent with the calculated value and resulting in increased phosphorescence proportion from 30.8% in DBF and 92.3% in DBFDBT to 97.2% in DBFDBTCZ (Fig. S6b, Tables 1 and S4, ESI†). So, the moderate Δ*E*_ST_ allows the efficient ISC transition and blocks the undesired RISC process.

**Fig. 2:**
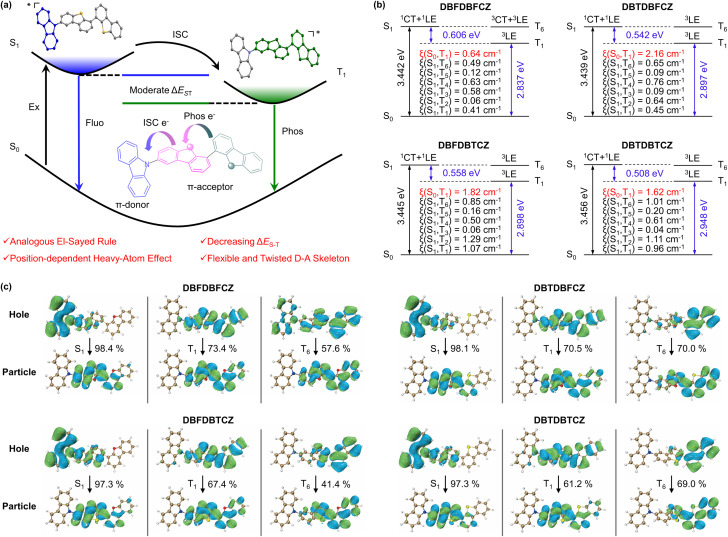
(a) Brief diagram describing multiple strategies. (b) The energy-level diagrams, molecular orbital characters, SOC coefficients (*ξ*), and (c) the natural transition orbitals of frontier excited singlet states and excited triplet states for DBFDBFCZ, DBTDBFCZ, DBFDBTCZ, and DBTDBTCZ in the monomeric state at the (TD)B3LYP/def2-SVP level.

Based on the aforementioned results, this study demonstrates synergistic strategies to boost phosphorescence in weakly D–A ternary π-conjugated skeletons (Fig. 2a). The flexible twisted skeleton follows an analogous El-Sayed rule, providing an additional pathway to improve ISC efficiency. The incorporation of heavy atoms at the excitation and emission region enhances the intersystem crossing and phosphorescence decay, respectively. Notably, the weak charge-transfer induced by the D–A structure in the excited state plays a vital role in reducing Δ*E*_ST_. Consequently, these factors lead to the enhancement of phosphorescence properties in ternary π-conjugated molecules, as depicted in Fig. 2a.

To gain a deeper insight into the photophysical process, we have simulated the transition processes and orbital properties of ternary π-conjugated molecules (Fig. 2b and c). From calculated natural transition orbits, it is evident that these molecules exhibit a distinct distribution of D–A characteristics with carbazole as the donor and binary skeletons as acceptors, resulting in the S_1_ with major ^1^CT and minor ^1^LE characteristics. Additionally, the T_1_ endows dominating ^3^LE characteristics with the (π,π*) configurations from binary skeletons, verified by the second scale lifetimes (Table S3, ESI†). The energy-level diagrams demonstrate that the introduction of D–A skeletons contributes to reducing Δ*E*_ST_ in both S_1_–T_*n*_ and S_1_–T_1_ (Fig. 2b and S3, ESI†). Furthermore, under weak electronic coupling, both S_1_ and close-lying T_*n*_ states contain LE and CT characteristics contributed by donor and acceptor π-segments. The hybridized configurations should allow the analogous El-Sayed rule and accelerate the ISC process *via* a second-order vibronic coupling mechanism, as well as the larger *ξ*(S_*n*_–T_*n*_) constants (Table S5, ESI†). Therefore, we observed evidence for *ξ*(S_2_–T_*n*_) and *ξ*(S_3_–T_*n*_) being greater than *ξ*(S_1_–T_*n*_) in the system, indicating the influence of the analogous El-Sayed rule. Multiple ISC transition channels of up to six are depicted for these ternary skeletons, compared to monomers CZ, DBF, and DBT (Fig. S3, ESI†). Moreover, the introduction of the heavy-atom sulphur increases spin–orbit coupling (SOC) constants in a position-dependent manner (Table S5, ESI†). From SOC element values of *ξ*(S_0_–T_1_), sulphur-containing molecules exhibit larger *ξ*(S_0_–T_1_) than DBFDBFCZ. In detail, *ξ*(S_1_–T_*n*_)s of DBFDBTCZ are generally larger than those of DBTDBFCZ as high as 1.29 cm^−1^, because sulphur atom is close to CZ in DBFDBTCZ. This captivating finding elucidates the different RTP performances between DBTDBFCZ and DBFDBTCZ from an accurate heavy-atom position effect on the intersystem crossing and phosphorescence process. In short, more ISC pathways, larger SOC coefficients and reduced Δ*E*_S_1_–T_1__ in the ternary systems contribute to high-efficiency ISC and phosphorescence. It should be noted that the weakly D–A effect can hardly cause obvious charge separation and could not obtain triplet excitons through the pathway of charge recombination in strong D–A systems (Fig. S12, ESI†).^46^

### Single-component white emission

White luminescence serves as a crucial display and lighting technology and plays a vital role in daily life.^47–50^ In contrast to multi-component systems,^51^ the single-component white luminescence offers distinct advantages of facile processing, non-phase separation, and structural diversity.^25^ However, realizing single-component purely organic systems for white light emission encounters significant challenges due to the required and balanced multiple emissive states.^52–57^ Several methodologies have been developed, including dual fluorescence hybridization,^54,55,57–62^ fluorescence phosphorescence mixing,^56,63–67^ dual phosphorescence integration,^26,68^ and clusteroluminescence.^69–72^ Among these strategies, fluorescence phosphorescence mixing draws considerable interest in the abundant photophysical processes.

**Fig. 3:**
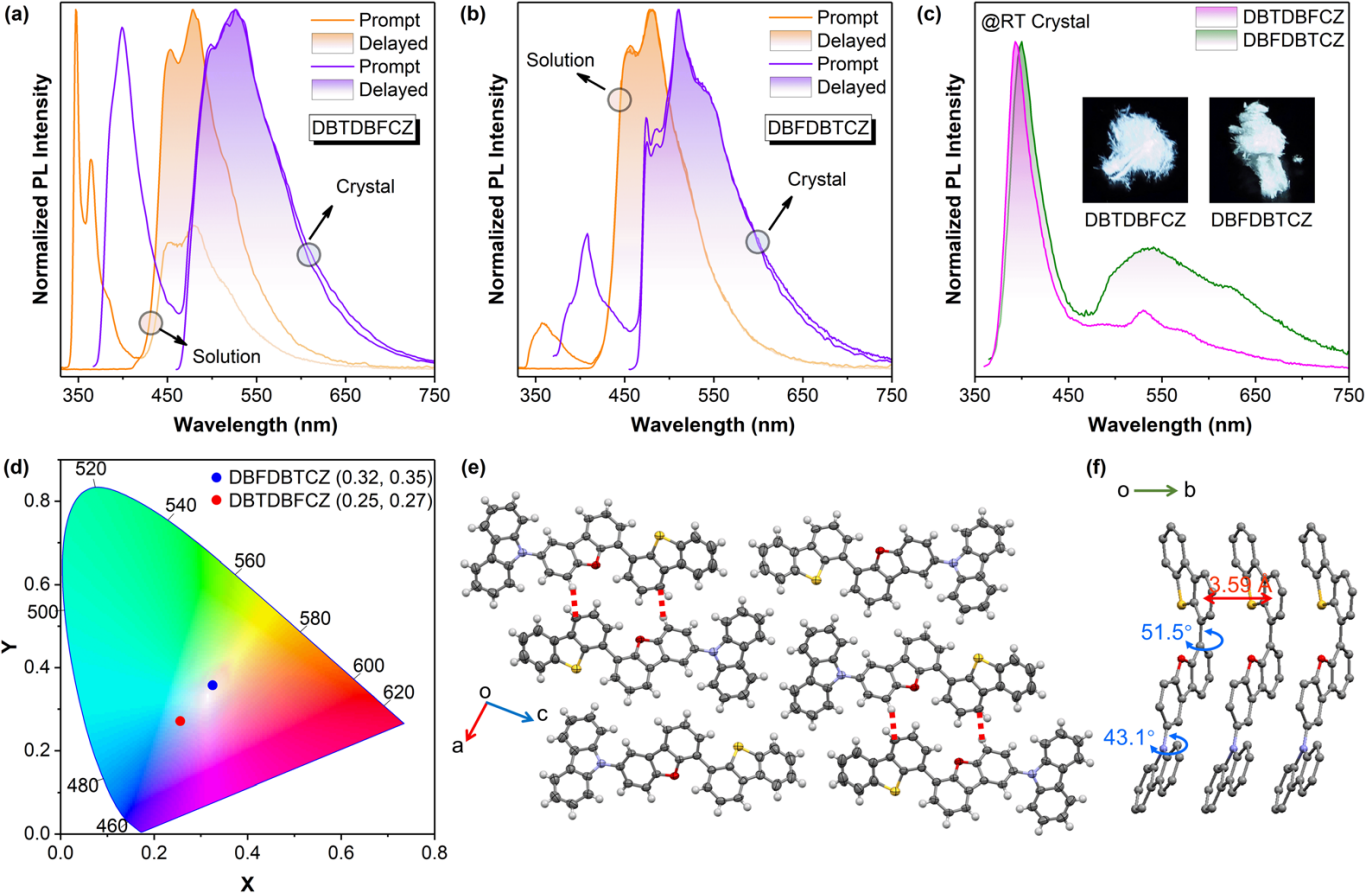
Normalized PL spectra and delayed (1 ms) spectra at 77 K in crystals and in 2-MeTHF (10^−5^ M) of (a) DBTDBFCZ and (b) DBFDBTCZ. (c) Normalized PL spectra at room temperature in crystals (insets: photographs of corresponding crystals taken under the UV excitation source of 365 nm under ambient conditions) and (d) corresponding CIE coordinates of DBTDBFCZ and DBFDBTCZ. (e) Crystal arrangement and (f) π–π packing of DBTDBFCZ.

The photophysical behaviours of DBTDBFCZ and DBFDBTCZ crystals were investigated. As shown in Fig. 3a, b and S7,† both fluorescence and phosphorescence spectra of crystals are redshifted and widened compared to those of solutions at 77 K. From the similar shape profiles and fluorescence-to-phosphorescence ratios, the phosphors in crystals inherited the emission properties in solutions. Intriguingly, it was found that DBTDBFCZ and DBFDBTCZ crystals emit distinct white luminescence at room temperature. Specifically, DBTDBFCZ yields cold white luminescence with Commission Internationale de l'Éclairage (CIE) 1931 coordinates of (0.25, 0.27), while DBFDBTCZ offers warm white luminescence with CIE 1931 coordinates of (0.32, 0.35) (Fig. 3c and d). The single-component white luminescence is mainly composed of blue fluorescence and yellow phosphorescence in a balance of luminescence wavelength and proportion. It is worth noting that the long wavelength proportion is contributed by crystallization-induced redshift of phosphorescence. In the case of DBFDBTCZ, the stronger intersystem crossing facilitates enhanced phosphorescence proportion, thereby enabling the warm white emission.

Further investigation into the crystal structure revealed distorted molecular configurations and π–π stacking with a face-to-face distance of 3.59 Å in DBTDBFCZ (Fig. 3e and f).^66^ The combination of the large torsion angle and π–π stacking further stabilizes the excited state, resulting in a redshift of the emission wavelength.^74^ However, upon closer analysis of the crystal cross-section, it becomes evident that the arrangement of molecules is staggered, and there is a limited occurrence of C–H⋯π interactions (Fig. 3e). The insufficient interactions allow flexible molecular twisting and rotation within the unit cell. Consequently, the dynamic relaxation of the excited states remains uncontrolled, leading to strong nonradiative decay of triplet excitons at room temperature in crystals.

### Doped PVA films and sensing

Controlling the external stimulus to regulate triplet emission represents a powerful approach for achieving functional RTP materials.^27,28,75^ The advantages of feasibly tunable afterglow lifetime, emission colour, and luminescence brightness of stimulus-responsive RTP materials thus benefit non-contact detection, *in situ* monitoring, and intuitive analysis with high contrast and sensitivity.^27^ On the other hand, polymers, possessing cross-linked matrices and adjustable side- and main-chain groups, have been found to be the ideal matrix for constructing stimulus-responsive RTP systems. Recently, transparent poly(vinyl alcohol) (PVA) with repeating 1,3-diol and 1,2-diol groups has emerged as a promising host matrix for establishing RTP film due to its sufficient hydroxyl groups that suppress intra- and intermolecular motions *via* the hydrogen bond networks.^73,76–80^

**Fig. 4:**
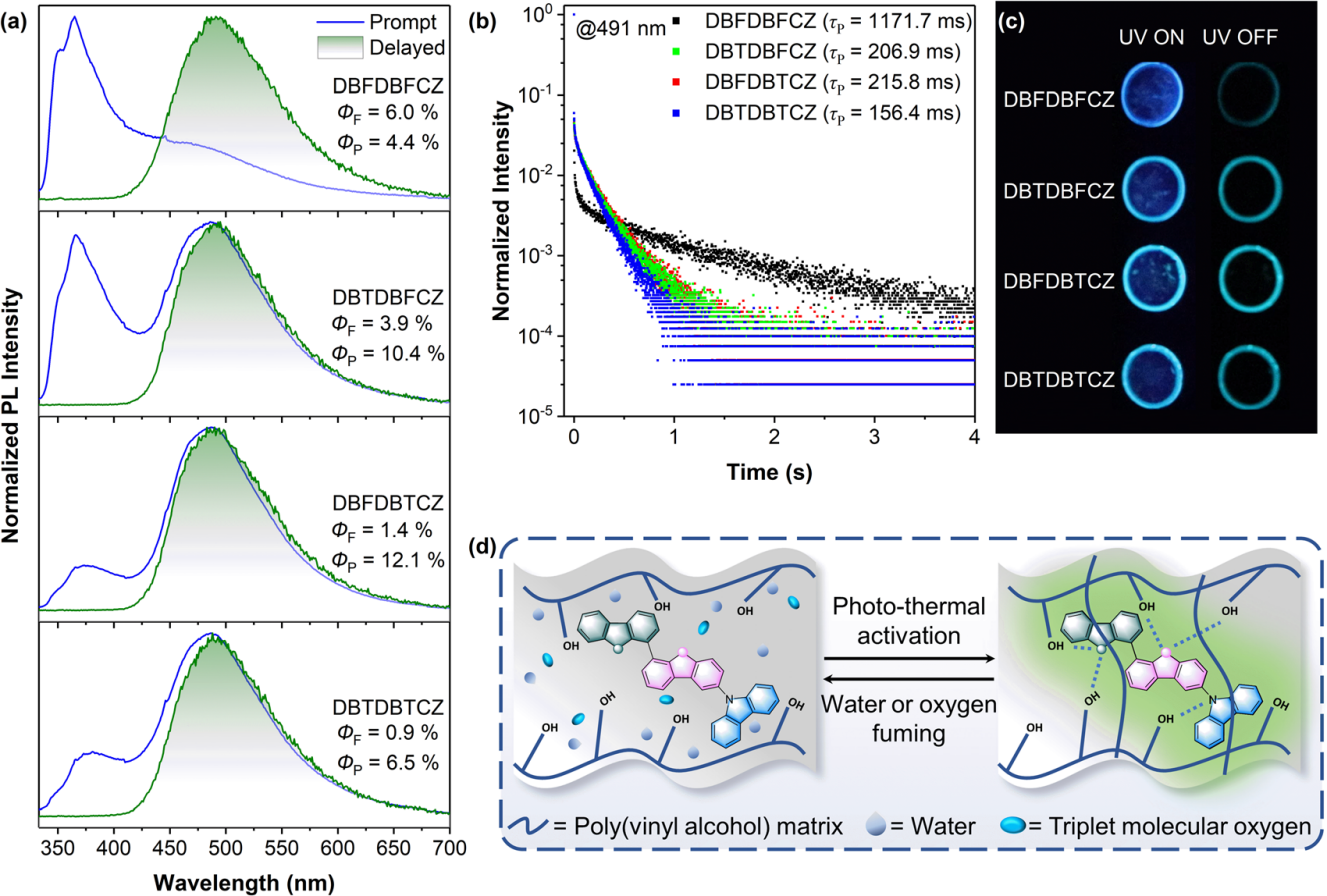
(a) Normalized PL spectra and delayed (1 ms) spectra at room temperature, (b) time-resolved phosphorescence decay curves at 491 nm, (c) photographs taken before and after the removal of the UV excitation source of 254 nm under ambient conditions of treated PVA films with doped DBFDBFCZ, DBTDBFCZ, DBFDBTCZ and DBTDBTCZ. (d) Schematic diagram of PVA film with doped ternary π-conjugated molecules before and after photo-thermal treatment and water or oxygen fuming.

In pursuit of intrinsic high-performance RTP, we prepared PVA films doped with ternary π-conjugated molecules at an optimized mass ratio of 1 : 1000. High concentration caused poor dispersion, and low concentration induced weak brightness and difficulty in detecting the RTP signal (Fig. S9, ESI†). Fig. S10† illustrates that as-prepared PVA films initially exhibit weak emission due to substantial interference from water and oxygen under ambient conditions. It was difficult to observe the phosphorescence with the naked eye and PL instruments (Fig. S10b, ESI†). To address these quenching effects, the films followed photo-thermal activation to remove the moisture and convert triplet oxygen into the singlet state (Fig. 4d). Remarkably, significant improvements in luminescence were observed post-treatment, resulting in enhanced quantum yields and afterglow characteristics.

As shown in Fig. 4a, the treated films demonstrated similar unimolecular emission behaviour to those observed in their low-temperature solutions. Notably, the phosphorescence in these films originated from the π-binary skeletons, and the proportion of phosphorescence was gradually increased. The reduction in fluorescence and the simultaneous improvement in phosphorescence from the DBFDBFCZ film to the DBFDBTCZ film highlight the heavy atom effects. Consistent afterglow wavelengths but different lifetimes and brightnesses were found (Fig. 4b and c). The photophysical parameters of these films are summarized in Table S6.† Notably, DBTDBTCZ film exhibited the maximum intersystem crossing rate (*k*_ISC_) and nonradiative rate (*k*_NR_), and the 1.7-fold larger *k*_ISC_ value for DBFDBTCZ@PVA compared to DBTDBFCZ@PVA demonstrated the position-dependent heavy atom effect in the ISC process. Overall, DBFDBTCZ exhibited optimized RTP performance in treated PVA films.

**Fig. 5:**
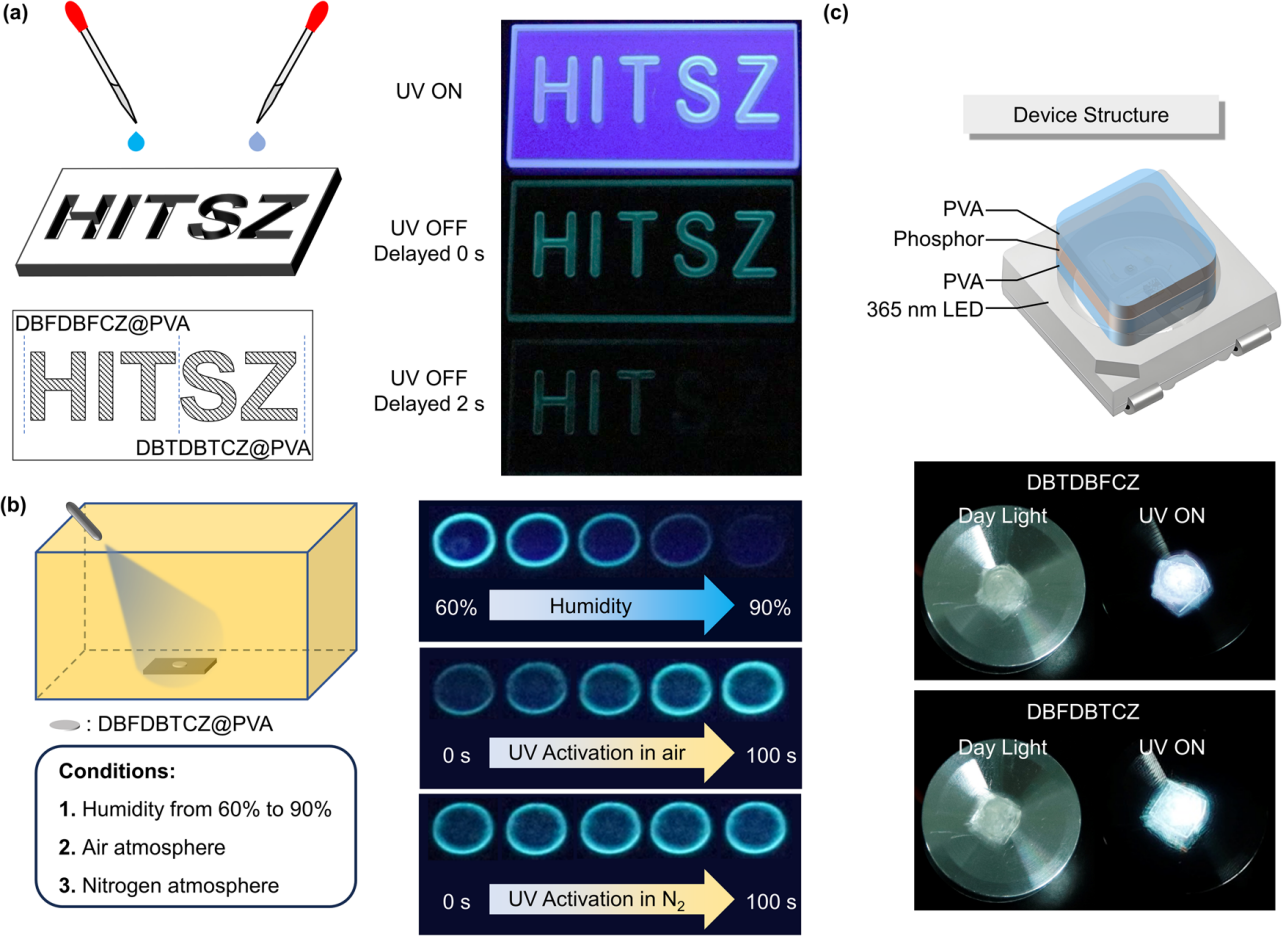
(a) Schematic diagram of the preparation of anti-counterfeiting patterns and photographs of the security code before and after turning off the 254 nm UV lamp. (b) Schematic diagram of environmental simulation under different humidities and oxygen atmosphere and photographs of the sensing process under 254 nm UV lamp excitation. (c) Schematic diagram in the preparation of a luminescence device, and photographs of devices under daylight and glowing of the device in operation.

Given the excellent phosphorescence performance of ternary π-conjugated molecules in PVA films, we developed their anti-counterfeiting and sensing applications. As depicted in Fig. 5a, the mould containing the “HIT” motif was filled with a DBFDBFCZ@PVA solution, while the “SZ” motif was filled with a DBTDBTCZ@PVA solution. After solvent evaporation and subsequent photo-thermal treatment, a bright luminescent pattern was observed upon UV excitation. Intriguingly, when the excitation ceased, a blue-green afterglow emerged. After two seconds, the afterglow associated with the “SZ” motif vanished, whereas the afterglow from the “HIT” motif remained. This unique combination of single emission colour and varying luminescence retention times sets this system different from traditional multi-colour afterglow patterns, rendering it rare and difficult to imitate and making them promising for anti-counterfeiting applications.

Besides, ternary π-conjugated molecules offer a direct and efficient approach for detecting humidity and oxygen levels by monitoring real-time PL brightness. The DBFDBTCZ-doped PVA films were photothermally activated before moisture and oxygen sensing. As shown in Fig. 5b, the emission brightness gradually decreased as the environmental humidity increased from 60% to 90%, with luminescence completely quenching at 90% humidity. The correlation between humidity and phosphorescence emission enables an intuitive assessment of approximate humidity levels without complex instruments. Remarkably, these films can be reverted to their initial state through re-photothermal treatment, confirming their repeatability and practicality. The oxygen sensing relies on a photoactivation process. The activated PVA films were placed in the air and they underwent an obvious and continuous enhancement of emission brightness upon UV excitation, lasting for 100 seconds. In contrast, when placed in nitrogen, the photoactivation effect was not observed and the film was always bright. This different photoactivation process confirms that the presence of triplet oxygen is detrimental to RTP emission and can be consumed by UV irradiation. The combination of real-time monitoring, fast responsiveness, and good repeatability makes ternary π-conjugated molecules valuable for humidity and oxygen detection with diverse practical implications in monitoring food spoilage or environmental change.

In particular, single-component luminophores with white light emission can maintain long-term emission without phase separation defects, making them ideal for lighting devices. With their solid-state dual emission characteristics, we conducted experiments to create white light devices. Initially, we applied the luminescent material as a drop-coated layer on the substrate, using a PVA film coating to reduce oxygen and moisture quenching. We selected a 365 nm LED lamp as the light source. The resulting device structure is depicted in Fig. 5c. Both devices demonstrated obvious white light emission, with the DBTDBFCZ device emitting cold white light and the DBFDBTCZ device emitting warm white light. These initial demonstrations highlight the potential applications of ternary conjugated molecules.

## Conclusions

In summary, this study proposed synergistic molecular strategies to construct efficient room-temperature phosphorescent systems. Through elaborate molecular design, we have developed a weakly donor–acceptor ternary π-conjugated framework, combining the analogous El-Sayed rule, halogen-atom-free heavy-atom effect, reduction of the singlet–triplet energy gap, and manipulation of flexible molecular conformation. The system successfully achieved a high phosphorescence-to-fluorescence ratio from 0.4 in carbazole to 35.2 in DBTDBTCZ. Simultaneously, with fine-tuned performance and co-planar π–π stacking, single-component white luminescence emitting warm and cool colours was achieved. Furthermore, we observed that position-dependent heavy atom effects selectively promoted intersystem crossing or increased phosphorescence decays. Ultimately, utilizing these highly efficient phosphorescence properties, we have accomplished real-time humidity monitoring, oxygen sensing, anti-counterfeiting labelling and white lighting. These promising applications inspire further investigation for establishing multifunctional and facilely prepared RTP functional materials.

## Data availability

The authors confirm that all the necessary data to support the findings of this study can be found within this article and its ESI.† The crystallographic data for this paper, including CCDC 2265477 for DBTDBFCZ and 2265478 for DBTDBTCZ, are available.

## Author contributions

W. H. performed all photophysical measurements, analysed the data, synthesized the materials, and grew the crystals. Y. Z. and L. S. performed the theoretical calculations. X. X. and G. T. assisted during the sample preparation. K. Z. assisted in the analysis of the molecular crystal structure. Z. H. designed and supervised the research and wrote the paper. All authors discussed the results and commented on the manuscript.

## Conflicts of interest

There are no conflicts to declare.

## Supplementary Material

SC-015-D4SC02525C-s001

SC-015-D4SC02525C-s002

## References

[cit1] Zhao W., He Z., Tang B. Z. (2020). Nat. Rev. Mater..

[cit2] Gu J., Li Z., Li Q. (2023). Coord. Chem. Rev..

[cit3] Zhou B., Yan D. (2023). Adv. Funct. Mater..

[cit4] Huang W., He Z. (2023). Synlett.

[cit5] Fateminia S. M. A., Mao Z., Xu S., Yang Z., Chi Z., Liu B. (2017). Angew. Chem., Int. Ed..

[cit6] Tian Z., Li D., Ushakova E. V., Maslov V. G., Zhou D., Jing P., Shen D., Qu S., Rogach A. L. (2018). Adv. Sci..

[cit7] Higginbotham H. F., Okazaki M., de Silva P., Minakata S., Takeda Y., Data P. (2021). ACS Appl. Mater. Interfaces.

[cit8] Gan N., Zou X., Dong M., Wang Y., Wang X., Lv A., Song Z., Zhang Y., Gong W., Zhao Z., Wang Z., Zhou Z., Ma H., Liu X., Chen Q., Shi H., Yang H., Gu L., An Z., Huang W. (2022). Nat. Commun..

[cit9] Shao W., Kim J. (2022). Acc. Chem. Res..

[cit10] Shi H., Yao W., Ye W., Ma H., Huang W., An Z. (2022). Acc. Chem. Res..

[cit11] Carretero A. S., Castillo A. S., Gutiérrez A. F. (2005). Crit. Rev. Anal. Chem..

[cit12] He Y., Wang J., Li Q., Qu S., Zhou C., Yin C., Ma H., Shi H., Meng Z., An Z. (2023). Adv. Opt. Mater..

[cit13] Xiong Z., Gong W., Xu P., Jiang M., Cai X., Zhu Y., Ping X., Feng H., Ma H., Qian Z. (2023). Chem. Eng. J..

[cit14] Yan Z.-A., Ma X. (2022). ACS Mater. Lett..

[cit15] Wu B., Su H., Cheng A., Zhang X., Wang T., Zhang G. (2023). Cell Rep. Phys. Sci..

[cit16] Zhao W., He Z., Lam J. W. Y., Peng Q., Ma H., Shuai Z., Bai G., Hao J., Tang B. Z. (2016). Chem.

[cit17] Ceroni P. (2016). Chem.

[cit18] Ma H., Peng Q., An Z., Huang W., Shuai Z. (2018). J. Am. Chem. Soc..

[cit19] Zhou Y., Qu L., Yi S., Wang C., Chen X., Tang S., Tang H., Li Y., Wang K., Zhao Y., Yang C. (2022). Adv. Opt. Mater..

[cit20] Lv X., Cao X., Wu H., Lin H., Ni F., Huang H., Zou Y., Yang C. (2021). Chem. Eng. J..

[cit21] Zhao J., Yan G., Wang W., Shao S., Yuan B., Li Y. J., Zhang X., Huang C. Z., Gao P. F. (2022). Research.

[cit22] Huang W., Zhu Y., Zhou K., Chen L., Zhao Z., Zhao E., He Z. (2024). Chem.–Eur. J..

[cit23] Garain S., Sarkar S., Chandra Garain B., Pati S. K., George S. J. (2022). Angew. Chem., Int. Ed..

[cit24] Su H., Hu K., Huang W., Wang T., Zhang X., Chen B., Miao H., Zhang X., Zhang G. (2023). Angew. Chem., Int. Ed..

[cit25] Zhang J., Zhao X., Shen H., Lam J. W. Y., Zhang H., Tang B. Z. (2021). Adv. Photonics.

[cit26] He Z., Zhao W., Lam J. W. Y., Peng Q., Ma H., Liang G., Shuai Z., Tang B. Z. (2017). Nat. Commun..

[cit27] Yang J., Fang M., Li Z. (2020). InfoMat.

[cit28] Lei Y., Dai W., Li G., Zhang Y., Huang X., Cai Z., Dong Y. (2023). J. Phys. Chem. Lett..

[cit29] Xie W., Huang W., Li J., He Z., Huang G., Li B. S., Tang B. Z. (2023). Nat. Commun..

[cit30] Li X., Shen S., Zhang C., Liu M., Lu J., Zhu L. (2021). Sci. China: Chem..

[cit31] Etherington M. K., Gibson J., Higginbotham H. F., Penfold T. J., Monkman A. P. (2016). Nat. Commun..

[cit32] Pokhilko P., Krylov A. I. (2019). J. Phys. Chem. Lett..

[cit33] Wu Z., Nitsch J., Schuster J., Friedrich A., Edkins K., Loebnitz M., Dinkelbach F., Stepanenko V., Würthner F., Marian C. M., Ji L., Marder T. B. (2020). Angew. Chem., Int. Ed..

[cit34] Zhou J., Stojanović L., Berezin A. A., Battisti T., Gill A., Kariuki B. M., Bonifazi D., Crespo-Otero R., Wasielewski M. R., Wu Y.-L. (2021). Chem. Sci..

[cit35] Yan Z.-A., Lin X., Sun S., Ma X., Tian H. (2021). Angew. Chem., Int. Ed..

[cit36] Isse A. A., Gennaro A., Lin C. Y., Hodgson J. L., Coote M. L., Guliashvili T. (2011). J. Am. Chem. Soc..

[cit37] Ma J., Zhang X., Phillips D. L. (2019). Acc. Chem. Res..

[cit38] Matsuo K., Yamaguchi E., Itoh A. (2023). J. Org. Chem..

[cit39] Juliá F., Constantin T., Leonori D. (2022). Chem. Rev..

[cit40] Song J., Wang Y., Qu L., Fang L., Zhou X., Xu Z.-X., Yang C., Wu P., Xiang H. (2022). J. Phys. Chem. Lett..

[cit41] Chen C., Chi Z., Chong K. C., Batsanov A. S., Yang Z., Mao Z., Yang Z., Liu B. (2021). Nat. Mater..

[cit42] Minaev B., Baryshnikov G., Agren H. (2014). Phys. Chem. Chem. Phys..

[cit43] Yu L., Wu Z., Zhong C., Xie G., Zhu Z., Ma D., Yang C. (2017). Adv. Opt. Mater..

[cit44] Chen C., Huang R., Batsanov A. S., Pander P., Hsu Y.-T., Chi Z., Dias F. B., Bryce M. R. (2018). Angew. Chem., Int. Ed..

[cit45] Kukhta N. A., Huang R., Batsanov A. S., Bryce M. R., Dias F. B. (2019). J. Phys. Chem. C.

[cit46] Gillett A. J., Privitera A., Dilmurat R., Karki A., Qian D., Pershin A., Londi G., Myers W. K., Lee J., Yuan J., Ko S.-J., Riede M. K., Gao F., Bazan G. C., Rao A., Nguyen T.-Q., Beljonne D., Friend R. H. (2021). Nature.

[cit47] Lin H., Hu T., Cheng Y., Chen M., Wang Y. (2018). Laser Photonics Rev..

[cit48] Pan M., Liao W.-M., Yin S.-Y., Sun S.-S., Su C.-Y. (2018). Chem. Rev..

[cit49] Zhang Q.-W., Li D., Li X., White P. B., Mecinović J., Ma X., Ågren H., Nolte R. J. M., Tian H. (2016). J. Am. Chem. Soc..

[cit50] Wang C., Li Y., Lv Q., Zheng H., Zhu G., Xu X., Wang Y. (2022). Chem. Eng. J..

[cit51] Chen Z., Ho C.-L., Wang L., Wong W.-Y. (2020). Adv. Mater..

[cit52] Ma L., Ma X. (2022). Sci. China: Chem..

[cit53] Kasha M. (1950). Discuss. Faraday Soc..

[cit54] Tang K.-C., Chang M.-J., Lin T.-Y., Pan H.-A., Fang T.-C., Chen K.-Y., Hung W.-Y., Hsu Y.-H., Chou P.-T. (2011). J. Am. Chem. Soc..

[cit55] Li B., Li Z., Guo F., Song J., Jiang X., Wang Y., Gao S., Wang J., Pang X., Zhao L., Zhang Y. (2020). ACS Appl. Mater. Interfaces.

[cit56] Wang J., Gu X., Ma H., Peng Q., Huang X., Zheng X., Sung S. H. P., Shan G., Lam J. W. Y., Shuai Z., Tang B. Z. (2018). Nat. Commun..

[cit57] Samanta S., Manna U., Das G. (2017). New J. Chem..

[cit58] Liu H., Cheng X., Zhang H., Wang Y., Zhang H., Yamaguchi S. (2017). Chem. Commun..

[cit59] Chen Y., Fang Y., Gu H., Qiang J., Li H., Fan J., Cao J., Wang F., Lu S., Chen X. (2020). ACS Appl. Mater. Interfaces.

[cit60] Chatsirisupachai J., Nalaoh P., Kaiyasuan C., Chasing P., Sudyoadsuk T., Promarak V. (2021). Mater. Chem. Front..

[cit61] Chen X., Yang Z., Li W., Mao Z., Zhao J., Zhang Y., Wu Y.-C., Jiao S., Liu Y., Chi Z. (2019). ACS Appl. Mater. Interfaces.

[cit62] Feng X., Qi C., Feng H.-T., Zhao Z., Sung H. H. Y., Williams I. D., Kwok R. T. K., Lam J. W. Y., Qin A., Tang B. Z. (2018). Chem. Sci..

[cit63] Wen Y., Liu H., Zhang S.-T., Pan G., Yang Z., Lu T., Li B., Cao J., Yang B. (2020). CCS Chem..

[cit64] Xu B., Wu H., Chen J., Yang Z., Yang Z., Wu Y.-C., Zhang Y., Jin C., Lu P.-Y., Chi Z. (2017). Chem. Sci..

[cit65] Cai S., Shi H., Tian D., Ma H., Cheng Z., Wu Q., Gu M., Huang L., An Z., Peng Q., Huang W. (2018). Adv. Funct. Mater..

[cit66] Wen Y., Liu H., Zhang S., Gao Y., Yan Y., Yang B. (2019). J. Mater. Chem. C.

[cit67] Zhou C., Zhang S., Gao Y., Liu H., Shan T., Liang X., Yang B., Ma Y. (2018). Adv. Funct. Mater..

[cit68] Behera S. K., Kainda R., Basu S., Chaudhary Y. S. (2022). Appl. Mater. Today.

[cit69] Zhou Z., Mao Z., Yang Z., Yang T., Zhu L., Long Y., Chi Z., Liu S., Aldred M. P., Chen X., Xu J., Zhang Y. (2021). Sci. China: Chem..

[cit70] Liu B., Chen Z., Chu B., Wang Y.-L., Li N., Zhang H., Yang Y., Hu S., Zhang X.-H. (2021). Adv. Photonics Res..

[cit71] Shang C., Zhao Y., Long J., Ji Y., Wang H. (2020). J. Mater. Chem. C.

[cit72] Liu B., Wang Y.-L., Bai W., Xu J.-T., Xu Z.-K., Yang K., Yang Y.-Z., Zhang X.-H., Du B.-Y. (2017). J. Mater. Chem. C.

[cit73] Zhou Y., Qin W., Du C., Gao H., Zhu F., Liang G. (2019). Angew. Chem., Int. Ed..

[cit74] Wu G., Li F., Tang B., Zhang X. (2022). J. Am. Chem. Soc..

[cit75] Gu F., Ma X. (2022). Chem.–Eur. J..

[cit76] Xiong S., Xiong Y., Wang D., Pan Y., Chen K., Zhao Z., Wang D., Tang B. Z. (2023). Adv. Mater..

[cit77] Yao X., Ma H., Wang X., Wang H., Wang Q., Zou X., Song Z., Jia W., Li Y., Mao Y., Singh M., Ye W., Liang J., Zhang Y., Liu Z., He Y., Li J., Zhou Z., Zhao Z., Zhang Y., Niu G., Yin C., Zhang S., Shi H., Huang W., An Z. (2022). Nat. Commun..

[cit78] Wu H., Chi W., Chen Z., Liu G., Gu L., Bindra A. K., Yang G., Liu X., Zhao Y. (2019). Adv. Funct. Mater..

[cit79] Huang W., Fu C., Liang Z., Zhou K., He Z. (2022). Angew. Chem., Int. Ed..

[cit80] Liang Z., Wei M., Zhang S., Huang W., Shi N., Lv A., Ma H., He Z. (2023). ACS Appl. Mater. Interfaces.

