# Supplementary material for: Utilizing weakly donor–acceptor ternary π-conjugated architecture to achieve single-component white luminescence and stimulus-responsive room-temperature phosphorescence
Source: Chem Sci. 2024 Jul 5;15(31):12316–25. doi: 10.1039/d4sc02525c (PMC11304527; doi:10.1039/d4sc02525c)
Supplement: SC-015-D4SC02525C-s001 [file SC-015-D4SC02525C-s001.pdf]

Electronic Supplementary Material (ESI) for Chemical Science.
This journal is © The Royal Society of Chemistry 2024

Electronic Supplementary Material (ESI) for Chemical Science
The journal is © The Royal Society of Chemistry 2024

Supporting information

Utilizing Weakly Donor-Acceptor Ternary π-Conjugated Architecture to Achieve Single-Component White Luminescence and Stimulus-Response Room-temperature Phosphorescence

Wenbin Huang,[a+] Yuxin Zhu,[a+] Xinwei Xie,[b] Guanqun Tang,[b] Kang Zhou[c], Lijuan Song,*[a] and Zikai He*[a]

a. School of Science, Harbin Institute of Technology Shenzhen, Shenzhen, Guangdong 518055, China.
b. School of Civil and Environmental Engineering, Harbin Institute of Technology Shenzhen, Shenzhen, Guangdong 518055, China.
c. Hoffman Institute of Advanced Materials, Shenzhen Polytechnic, Shenzhen, Guangdong 518055, China.

[+] These authors contributed equally.

General experimental details

All reactions were carried out under a nitrogen atmosphere. Starting materials and solvents were used without treatment. ^{1}H and ^{13}C spectra were carried out on QOne Instruments Quantum-I 400 M, and all NMR data was processed in MestReNova. All compounds were further purified by circulating liquid gel permeation chromatography with JAI LC-5060 equipped with JAIGEL-2.5HR and JAIGEL-2HR. UV-visible spectra were collected on Shimadzu UV-2600. High-resolution mass spectroscopy was carried out on Waters ACQUITYUPLC H-Class/Xevo G2-XS QTof. Single-crystal XRD was carried out on Rigaku XtaLAB Synergy and Bruker APEX-II CCD. All photophysical measurements, including steady or delay spectra, decay curve, and quantum yield, were carried out on Edinburgh Instruments FLS1000 with different accessories. High-performance liquid chromatography was carried out on Agilent 1260 LC equipped with WondaSil C18 Superb 5 μm 4.6 × 250 mm.

Synthesis and characterization

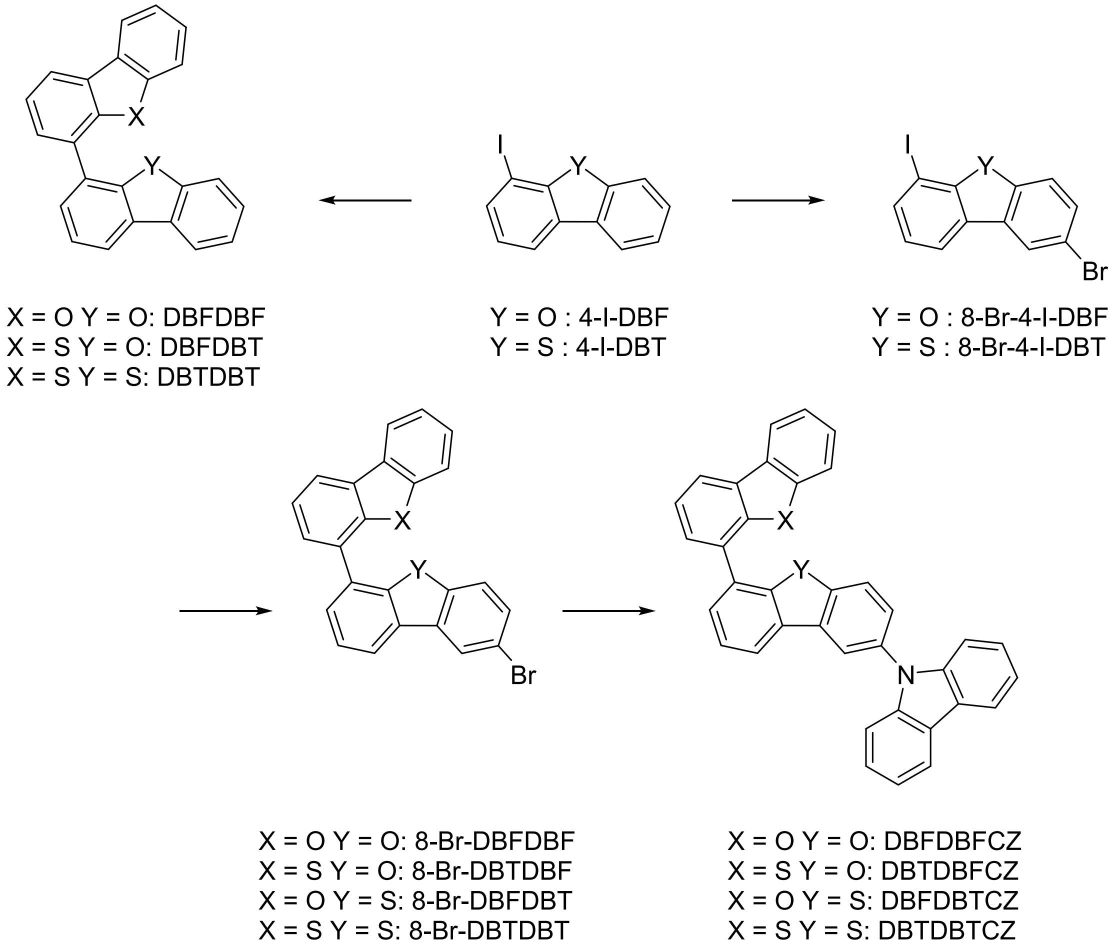

Scheme S1. Synthetic routes for compounds, together with labels for segments.

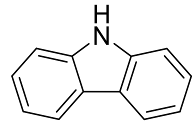

Carbazole

A 150 mL flask was charged with 2-bromodiphenylamine (3.9699 g, 16 mmol), $Pd(OAc)_2$ (0.1078 g, 0.48 mmol), $PCy_3{\cdot}HBF_4$ (0.3535 g, 0.96 mmol) and K_2CO_3 (4.4227 g, 32 mmol) under nitrogen atmosphere. 80 mL DMAc were injected into the flask maintaining 403 K under stirring for 10 hours. Afterwards, a saturated solution of NaCl was added, and the mixture was extracted with dichloromethane three times. The combined organic layer was dried over sodium sulfate. The solvent was removed under reduced pressure. The residue was purified by silica gel column chromatography using PE/DCM = 1/3 as an eluent. Yield: 60.2 % (1.6106 g).

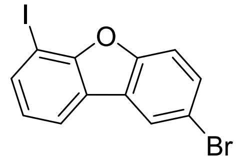

8-Br-4-I-DBF

A 25 mL flask was charged with 4-iododibenzofuran (0.8823 g, 3 mmol) and 7 mL acetic acid, and the resulting mixture was maintaining 333 K to dissolve completely. Subsequently, 0.2 mL liquid bromine was injected into the flask and then temperature returned to 313 K to keep stirring for 40 hours. Afterwards, the solvent was quenched by saturated sodium sulfite solution and was under filtration. Finally, the filter residue was recrystallization repeatedly for further purification. Yield: 17.9 % (0.2003 g).

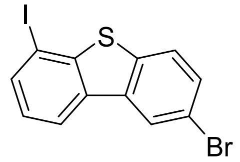

8-Br-4-I-DBT

A 25 mL flask was charged with 4-iododibenzothiophene (0.9305 g, 3 mmol) and 14 mL acetic acid and the resulting mixture was maintaining 343 K to dissolve completely. Subsequently, 0.2 mL liquid bromine was injected into the flask and then temperature returned to 313 K to keep stirring for 70 hours. Afterwards, the solvent was quenched by saturated sodium sulfite solution and was under filtration. Finally, the filter residue was recrystallization repeatedly for further purification. Yield: 21.3 % (0.2486 g).

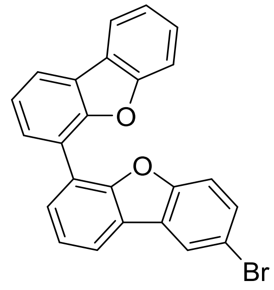

8-Br-DBFDBF

A 25 mL flask was charged with 8-bromo-4-iododibenzofuran (0.3730 g, 1 mmol), dibenzofuran-4-ylboronic acid (0.2332 g, 1.1 mmol), Tetrakis(triphenylphosphine)palladium (0.0693 g, 0.06 mmol), potassium carbonate (0.3041 g, 2.2 mmol) under nitrogen atmosphere. Degassing water 2.25 mL, ethyl alcohol 2.25 mL, toluene 10.5 mL were injected into the flask and the resulting mixture was maintaining 383 K and for 6 hours. Subsequently, it was extracted with dichloromethane and rotary evaporated to obtain residue for purified by silica-gel column chromatography using petroleum ether as the eluent, followed by recrystallization for further purification. Yield: 74.7% (0.3087 g).

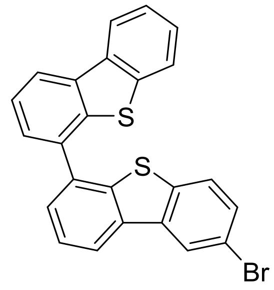

8-Br-DBTDBT

A 25 mL flask was charged with 8-bromo-4-iododibenzothiophene (0.3890 g, 1 mmol), dibenzothiophen-4-ylboronic acid (0.2509 g, 1.1 mmol), Tetrakis(triphenylphosphine)palladium (0.0693 g, 0.06 mmol), potassium carbonate (0.3041 g, 2.2 mmol) under nitrogen atmosphere. Degassing water 2.25 mL, ethyl alcohol 2.25 mL, toluene 10.5 mL were injected into the flask and the resulting mixture was maintaining 383 K and for 6 hours. Subsequently, it was extracted with dichloromethane and rotary evaporated to obtain residue for purified by silica-gel column chromatography using petroleum ether as the eluent, followed by recrystallization for further purification. Yield: 91.4% (0.4071 g).

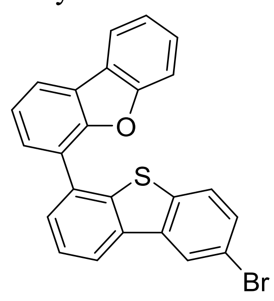

8-Br-DBFDBT

A 25 mL flask was charged with 8-bromo-4-iododibenzothiophene (0.3890 g, 1 mmol), dibenzofuran-4-ylboronic acid (0.2332 g, 1.1 mmol),

Tetrakis(triphenylphosphine)palladium (0.0693 g, 0.06 mmol), potassium carbonate (0.3041 g, 2.2 mmol) under nitrogen atmosphere. Degassing water 2.25 mL, ethyl alcohol 2.25 mL, toluene 10.5 mL were injected into the flask and the resulting mixture was maintaining 383 K and for 6 hours. Subsequently, it was extracted with dichloromethane and rotary evaporated to obtain residue for purified by silica-gel column chromatography using petroleum ether as the eluent, followed by recrystallization for further purification. Yield: 62.7% (0.2692 g).

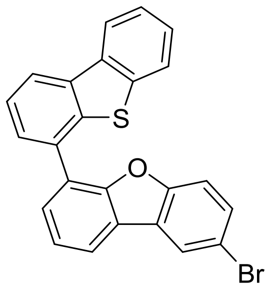

8-Br-DBTDBF

A 25 mL flask was charged with 8-bromo-4-iododibenzofuran (0.3730 g, 1 mmol), dibenzothiophen-4-ylboronic acid (0.2509 g, 1.1 mmol), Tetrakis(triphenylphosphine)palladium (0.0693 g, 0.06 mmol), potassium carbonate (0.3041 g, 2.2 mmol) under nitrogen atmosphere. Degassing water 2.25 mL, ethyl alcohol 2.25 mL, toluene 10.5 mL were injected into the flask and the resulting mixture was maintaining 383 K and for 6 hours. Subsequently, it was extracted with dichloromethane and rotary evaporated to obtain residue for purified by silica-gel column chromatography using petroleum ether as the eluent, followed by recrystallization for further purification. Yield: 37.4% (0.1606 g).

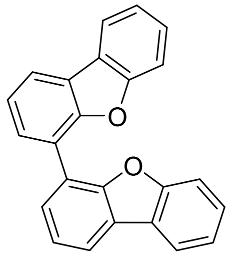

DBFDBF

A 25 mL flask was charged with 4-bromodibenzofuran (0.2471 g, 1 mmol), dibenzofuran-4-ylboronic acid (0.2332 g, 1.1 mmol), Tetrakis(triphenylphosphine)palladium (0.0693 g, 0.06 mmol), potassium carbonate (0.3041 g, 2.2 mmol) under nitrogen atmosphere. Degassing water 2.25 mL, ethyl alcohol 2.25 mL, toluene 10.5 mL were injected into the flask and the resulting mixture was maintaining 383 K and for 6 hours. Subsequently, it was extracted with dichloromethane and rotary evaporated to obtain residue for purified by silica-gel column chromatography using petroleum ether as the eluent, followed by recrystallization for further purification. Yield: 86.3% (0.2886 g). ^{1}H NMR (400 MHz, Chloroform-*d*) δ 8.06 – 8.03 (m, 4H), 8.00 (dd, *J* = 7.6, 1.2 Hz, 2H), 7.60 – 7.51 (m, 4H), 7.47 (td, *J* = 7.2, 1.2 Hz, 2H), 7.39 (td, *J* = 7.6, 1.2 Hz, 2H). ^{13}C NMR (101 MHz, Chloroform-*d*) δ 156.34, 153.78, 128.67, 127.37, 125.06, 124.42, 123.06, 122.91,

121.12, 120.84, 120.45, 112.02. HRMS (ESI) Calcd. For $C_{24}H_{15}O_2$ 335.1072, Found 335.1062 $[M+H]^+$. DBFDBF crystal: After the sample powder was dissolved in THF, petroleum ether was added slowly. As the petroleum ether slowly diffused into the bottom layer, single crystal was grown.

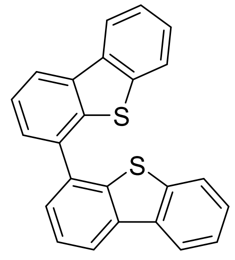

DBTDBT

A 25 mL flask was charged with 4-bromodibenzothiophene (0.2632 g, 1 mmol), dibenzothiophen-4-ylboronic acid (0.2509 g, 1.1 mmol), Tetrakis(triphenylphosphine)palladium (0.0693 g, 0.06 mmol), potassium carbonate (0.3041 g, 2.2 mmol) under nitrogen atmosphere. Degassing water 2.25 mL, ethyl alcohol 2.25 mL, toluene 10.5 mL were injected into the flask and the resulting mixture was maintaining 383 K and for 6 hours. Subsequently, it was extracted with dichloromethane and rotary evaporated to obtain residue for purified by silica-gel column chromatography using petroleum ether as the eluent, followed by recrystallization for further purification. Yield: 87.5% (0.3207 g). ^1H NMR (400 MHz, Chloroform-*d*) δ 8.26 (dd, J = 7.9, 1.1 Hz, 2H), 8.25 – 8.22 (m, 2H), 7.81 – 7.77 (m, 2H), 7.75 (dd, J = 7.4, 1.1 Hz, 2H), 7.64 (t, J = 7.6 Hz, 2H), 7.47 (pd, J = 7.2, 1.5 Hz, 4H). ^{13}C NMR (101 MHz, Chloroform-*d*) δ 139.69, 139.57, 136.58, 135.92, 135.40, 127.05, 126.94, 125.04, 124.56, 122.86, 121.91, 121.46. HRMS (ESI) Calcd. For $C_{24}H_{15}S_2$ 367.0615, Found 367.0638 $[M+H]^+$. DBTDBT crystal: After the sample powder was dissolved in THF, petroleum ether was added slowly. As the petroleum ether slowly diffused into the bottom layer, single crystal was grown.

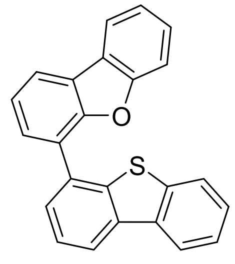

DBFDBT

A 25 mL flask was charged with 4-bromodibenzothiophene (0.2632 g, 1 mmol), dibenzofuran-4-ylboronic acid (0.2332 g, 1.1 mmol), Tetrakis(triphenylphosphine)palladium (0.0693 g, 0.06 mmol), potassium carbonate (0.3041 g, 2.2 mmol) under nitrogen atmosphere. Degassing water 2.25 mL, ethyl alcohol 2.25 mL, toluene 10.5 mL were injected into the flask and the resulting mixture was maintaining 383 K and for 6 hours. Subsequently, it was extracted with dichloromethane and rotary evaporated to obtain residue for purified by silica-gel column chromatography using petroleum ether as the eluent, followed by

recrystallization for further purification. Yield: 80.7% (0.2828 g). ^{1}H NMR (400 MHz, Chloroform-*d*) δ 8.30 – 8.20 (m, 2H), 8.09 – 8.01 (m, 2H), 7.88 – 7.76 (m, 3H), 7.66 (t, *J* = 7.6 Hz, 1H), 7.57 – 7.42 (m, 5H), 7.38 (t, *J* = 7.2 Hz, 1H). ^{13}C NMR (101 MHz, Chloroform-*d*) δ 156.31, 153.39, 151.84, 139.71, 136.46, 135.91, 131.66, 128.36, 127.49, 126.95, 125.17, 124.92, 124.84, 124.49, 124.37, 123.13, 122.98, 122.77, 121.87, 121.24, 120.87, 120.72, 112.09, 100.10. HRMS (ESI) Calcd. For $C_{24}H_{15}OS$ 351.0844, Found 351.0862 $[M+H]^+$. DBFDBT crystal: After the sample powder was dissolved in THF, petroleum ether was added slowly. As the petroleum ether slowly diffused into the bottom layer, single crystal was grown.

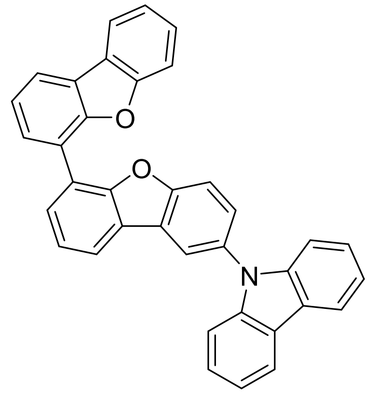

DBFDBFCZ

A 25 mL flask was charged with 8-Br-DBFDBF (0.4133 g, 1 mmol), carbazole (0.2007 g, 1.2 mmol), CuI (0.2857 g, 1.5 mmol), K_2CO_3 (0.4146 g, 3 mmol) under nitrogen atmosphere. 7 mL anhydrous DMAc was injected into the flask and the resulting mixture was maintaining 453 K and for 48 hours. Subsequently, the resulting solvent was quenched with 1 N saturated NaCl solution. Afterwards, the solvent was under filtration and the filter residue was purified by silica-gel column chromatography using dichloromethane/petroleum ether (1:4 v/v) as the eluent, followed by recrystallization for further purification. Yield: 49.5% (0.2473 g). ^{1}H NMR (400 MHz, Chloroform-*d*) δ 8.23 – 8.15 (m, 3H), 8.13 – 7.97 (m, 5H), 7.77 (d, *J* = 8.6 Hz, 1H), 7.66 – 7.53 (m, 4H), 7.53 – 7.47 (m, 1H), 7.47 – 7.38 (m, 5H), 7.32 (ddd, *J* = 8.0, 6.5, 1.6 Hz, 2H). ^{13}C NMR (101 MHz, Chloroform-*d*) δ 157.45, 156.40, 155.27, 154.62, 153.80, 141.69, 132.83, 129.43, 128.63, 127.48, 126.87, 126.14, 125.94, 125.16, 124.63, 124.41, 123.51, 123.38, 123.15, 123.01, 121.54, 120.91, 120.72, 120.67, 120.50, 120.02, 113.25, 112.05, 109.83. HRMS (ESI) Calcd. For $C_{36}H_{22}NO_2$ 500.1651, Found 500.1658 $[M+H]^+$. DBFDBFCZ crystal: After the sample powder was dissolved in THF, petroleum ether was added slowly. As the petroleum ether slowly diffused into the bottom layer, single crystal was grown.

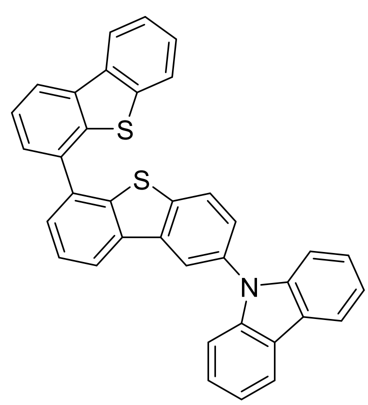

DBTDBTCZ

A 25 mL flask was charged with 8-Br-DBTDBT (0.4454 g, 1 mmol), carbazole (0.2007 g, 1.2 mmol), CuI (0.2857 g, 1.5 mmol), K_2CO_3 (0.4146 g, 3 mmol) under nitrogen atmosphere. 7 mL anhydrous DMAc was injected into the flask and the resulting mixture was maintaining 453 K and for 48 hours. Subsequently, the resulting solvent was quenched with 1 N saturated NaCl solution. Afterwards, the solvent was under filtration and the filter residue was purified by silica-gel column chromatography using dichloromethane/petroleum ether (1:4 v/v) as the eluent, followed by recrystallization for further purification. Yield: 37.6% (0.1999 g). ^{1}H NMR (400 MHz, Chloroform-*d*) (ppm): δ 8.40 (s, 2H), 8.30 (d, *J* = 7.9 Hz, 1H), 8.25 (d, *J* = 7.6 Hz, 1H), 8.21 (t, *J* = 8.3 Hz, 3H), 8.00 (d, *J* = 8.4, 1H), 7.85 – 7.75 (m, 3H), 7.66 (q, J = 9.8, 8.6 Hz, 3H), 7.53 – 7.48 (m, 2H), 7.44 (d, J = 3.8 Hz, 4H), 7.34 (dq, J = 7.9, 4.2 Hz, 2H). ^{13}C NMR (101 MHz, Chloroform-*d*) δ 143.87, 141.45, 140.49, 139.67, 139.58, 138.82, 137.51, 136.69, 136.09, 135.92, 135.71, 135.16 134.70, 127.61, 127.16, 126.93, 126.34, 126.20, 125.43, 125.14, 124.66, 124.24, 123.50, 122.91, 121.98, 121.79, 121.65, 120.76, 120.55, 120.15, 109.83. HRMS (ESI) Calcd. For $C_{36}H_{22}NS_2$ 532.1194, Found 532.1186 $[M+H]^+$. DBTDBTCZ crystal: After the sample powder was dissolved in THF, petroleum ether was added slowly. As the petroleum ether slowly diffused into the bottom layer, single crystal was grown.

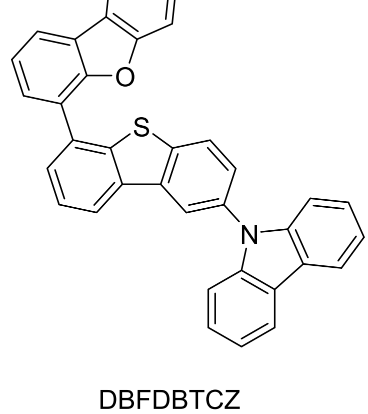

DBFDBTCZ

A 25 mL flask was charged with 8-Br-DBFDBT (0.4293 g, 1 mmol), carbazole (0.2007 g, 1.2 mmol), CuI (0.2857 g, 1.5 mmol), K_2CO_3 (0.4146 g, 3 mmol) under nitrogen

atmosphere. 7 mL anhydrous DMAc was injected into the flask and the resulting mixture was maintaining 453 K and for 48 hours. Subsequently, the resulting solvent was quenched with 1 N saturated NaCl solution. Afterwards, the solvent was under filtration and the filter residue was purified by silica-gel column chromatography using dichloromethane/petroleum ether (1:4 v/v) as the eluent, followed by recrystallization for further purification. Yield: 30.5% (0.1573 g). ^{1}H NMR (400 MHz, Chloroform-*d*) δ 8.40 (d, *J* = 1.6 Hz, 1H), 8.21 (t, *J* = 8.0 Hz, 3H), 8.09 (d, *J* = 7.7 Hz, 1H), 8.06 (d, *J* = 7.7 Hz, 1H), 8.02 (d, *J* = 8.4 Hz, 1H), 7.85 (d, *J* = 7.4 Hz, 2H), 7.71 – 7.62 (m, 2H), 7.58 – 7.52 (m, 2H), 7.51 – 7.43 (m, 5H), 7.40 (t, *J* = 7.4 Hz, 1H), 7.33 (dt, *J* = 8.0, 4.1 Hz, 2H). ^{13}C NMR (151 MHz, Chloroform-*d*) δ 153.41, 141.47, 138.88, 138.64, 137.49, 137.11, 135.95, 134.63, 132.03, 129.01, 127.61, 127.48, 126.24, 126.20, 125.92, 125.30, 124.61, 124.37, 124.15, 123.51, 123.37, 123.24, 123.09, 121.56, 120.94, 120.72, 120.55, 120.14, 112.13, 109.86. HRMS (ESI) Calcd. For $C_{36}H_{22}NOS$ 516.1422, Found 516.1436 $[M+H]^+$. DBFDBTCZ crystal: After the sample powder was dissolved in THF, petroleum ether was added slowly. As the petroleum ether slowly diffused into the bottom layer, single crystal was grown.

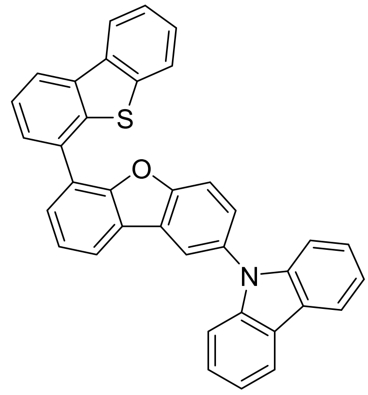

DBTDBFCZ

A 25 mL flask was charged with 8-Br-DBTDBF (0.4293 g, 1 mmol), carbazole (0.2007 g, 1.2 mmol), CuI (0.2857 g, 1.5 mmol), K_2CO_3 (0.4146 g, 3 mmol) under nitrogen atmosphere. 7 mL anhydrous DMAc was injected into the flask and the resulting mixture was maintaining 453 K and for 48 hours. Subsequently, the resulting solvent was quenched with 1 N saturated NaCl solution. Afterwards, the solvent was under filtration and the filter residue was purified by silica-gel column chromatography using dichloromethane/petroleum ether (1:4 v/v) as the eluent, followed by recrystallization for further purification. Yield: 36.2% (0.1867 g). ^{1}H NMR (400 MHz, Chloroform-*d*) δ 8.30 (d, *J* = 7.9 Hz, 1H), 8.26 (d, *J* = 7.2 Hz, 1H), 8.19 (d, *J* = 7.1 Hz, 3H), 8.04 (d, *J* = 7.7 Hz, 1H), 7.89 (d, *J* = 7.5 Hz, 1H), 7.83 (d, *J* = 7.1 Hz, 2H), 7.74 (d, *J* = 8.6 Hz, 1H), 7.70 (t, *J* = 7.6 Hz, 1H), 7.61 (d, *J* = 8.8 Hz, 1H), 7.52 (dt, *J* = 18.4, 6.9 Hz, 3H), 7.46 – 7.38 (m, 4H), 7.32 (t, *J* = 7.2 Hz, 2H). ^{13}C NMR (101 MHz, Chloroform-*d*) δ 155.24, 154.24, 148.82, 141.69, 139.80, 139.70, 136.55, 135.92, 132.91, 131.43, 128.35, 128.32, 127.07, 127.01, 126.15, 125.90, 125.22, 125.01, 124.75, 124.60, 123.59, 123.39, 122.82, 121.95, 121.02, 120.51, 120.04, 113.35, 109.81. HRMS (ESI) Calcd. For $C_{36}H_{22}NOS$ 516.1422, Found 516.1410 $[M+H]^+$. DBTDBFCZ crystal: After

the sample powder was dissolved in THF, petroleum ether was added slowly. As the petroleum ether slowly diffused into the bottom layer, single crystal was grown.

Treated film: 1 mg DBFDBFCZ was dissolved in 50 mL tetrahydrofuran to obtain a clarified guest solution by 10 minutes of ultrasound treatment. Similarly, 100 mg poly(vinyl alcohol) (PVA, 88 % hydrolyzed) was dissolved in 5 mL water to obtain clarified host solution by heating to 373 K. Then the 5 mL guest and 5 mL host solutions were mixed and ultrasonically treated for 10 minutes to prepare the mixed solution with a mass fraction ratio of 1:1000. Drop the mixed solution onto a square silicone plate with a side length of 2 cm and wait to dry at room temperature. The silicone plate was removed to obtain a transparent PVA film with doped DBFDBFCZ. As-prepared film was heated at 393 K for 30 minutes and then was excited by 254 nm ultraviolet light at room temperature for 10 minutes to obtain treated PVA film with doped DBFDBFCZ. Similarly, treated PVA films with doped DBFDBTCZ, DBTDBFCZ, and DBTDBTCZ are obtained by following this process.

Computing method: The ground state (S_0), the lowest singlet excited state (S_1) and the lowest triplet excited state (T_1) of the molecules at monomeric state are optimized at the (TD)B3LYP/def2-SVP level using the Gaussian 09 package. Based on the optimized geometry of S_1 and T_1, the natural transition orbits of S_1 and T_1 are calculated by Multiwfn. Corresponding spin-orbit coupling constant (ξ) is calculated at B3LYP/x2c-SVPall level by Beijing Density Function(BDF) Software.

^{1}H, ^{13}C NMR spectra of new compounds

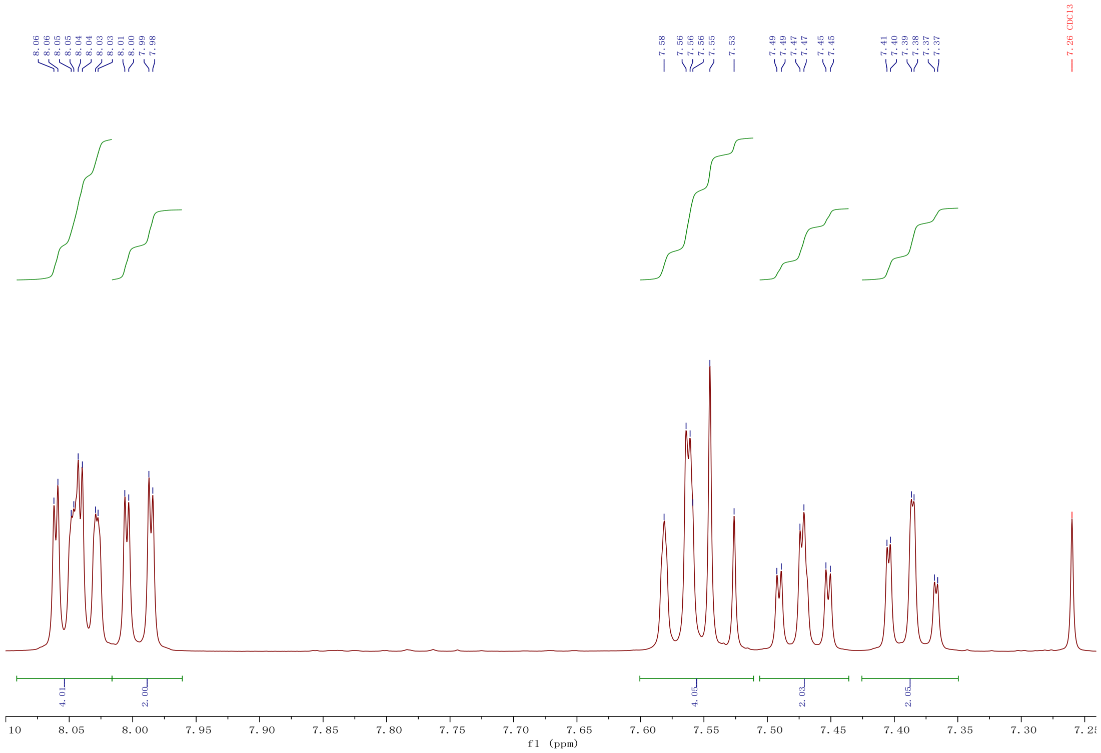

Chart S1 ^{1}H NMR spectrum of DBFDBF in Chloroform-*d*.

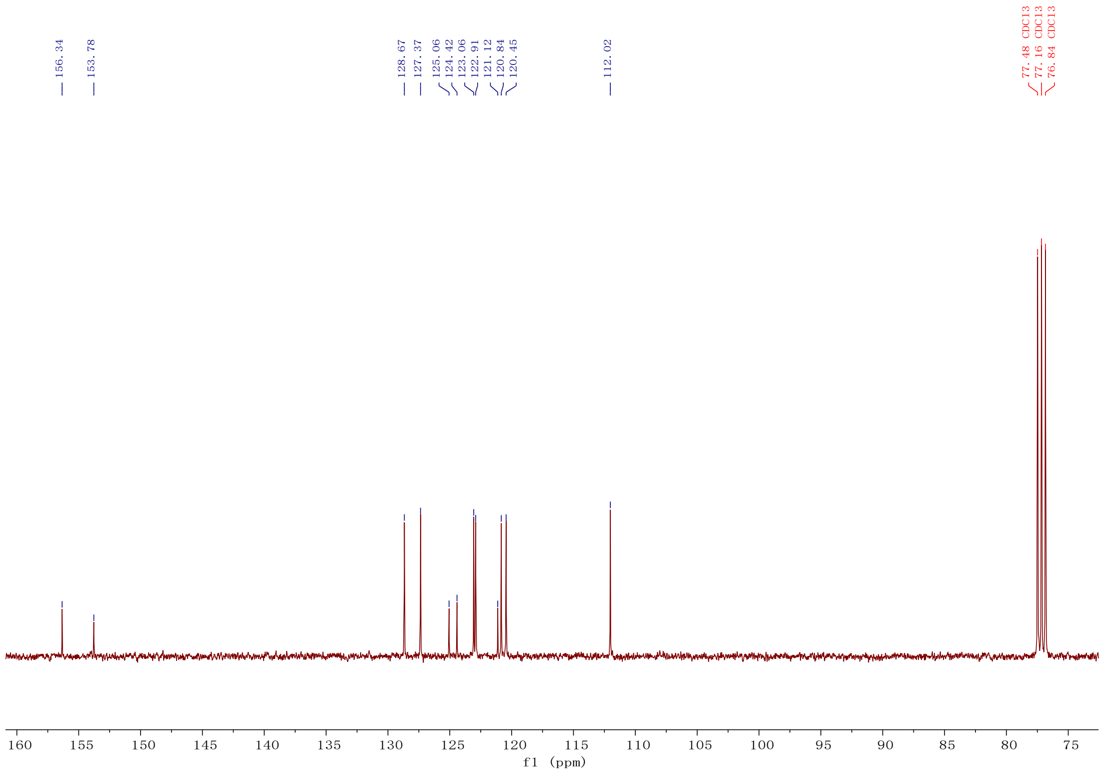

Chart S2 ^{13}C NMR spectrum of DBFDBF in Chloroform-*d*.

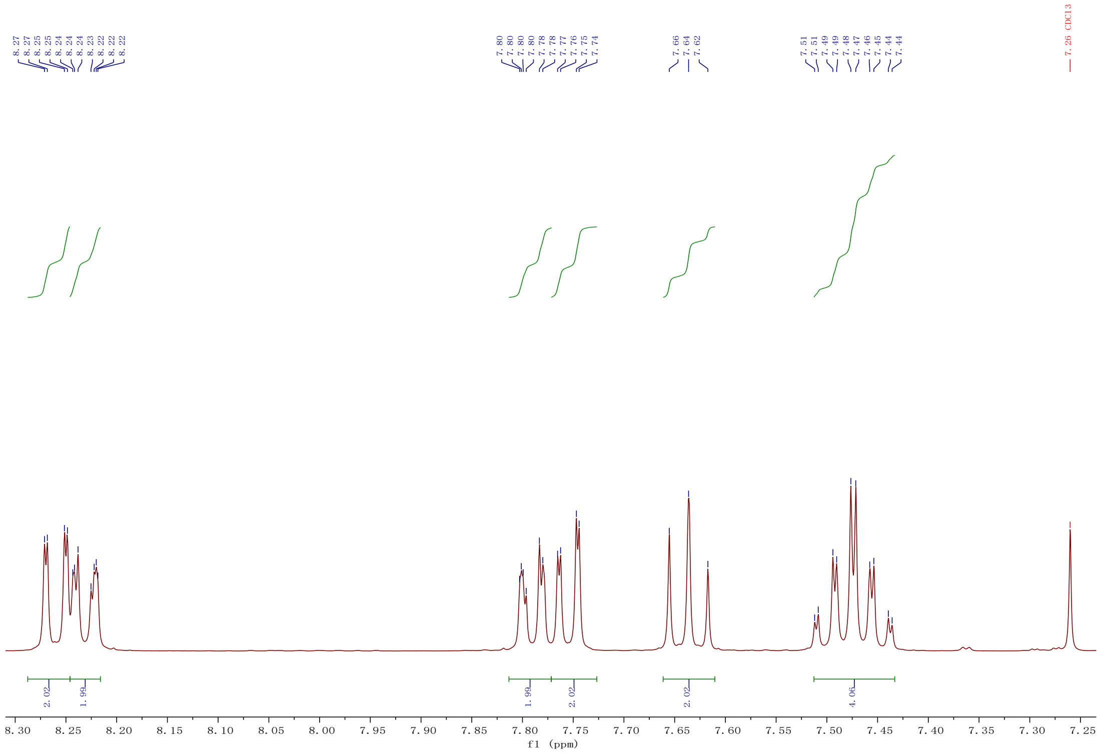

Chart S3 ^{1}H NMR spectrum of DBTDBT in Chloroform-*d*.

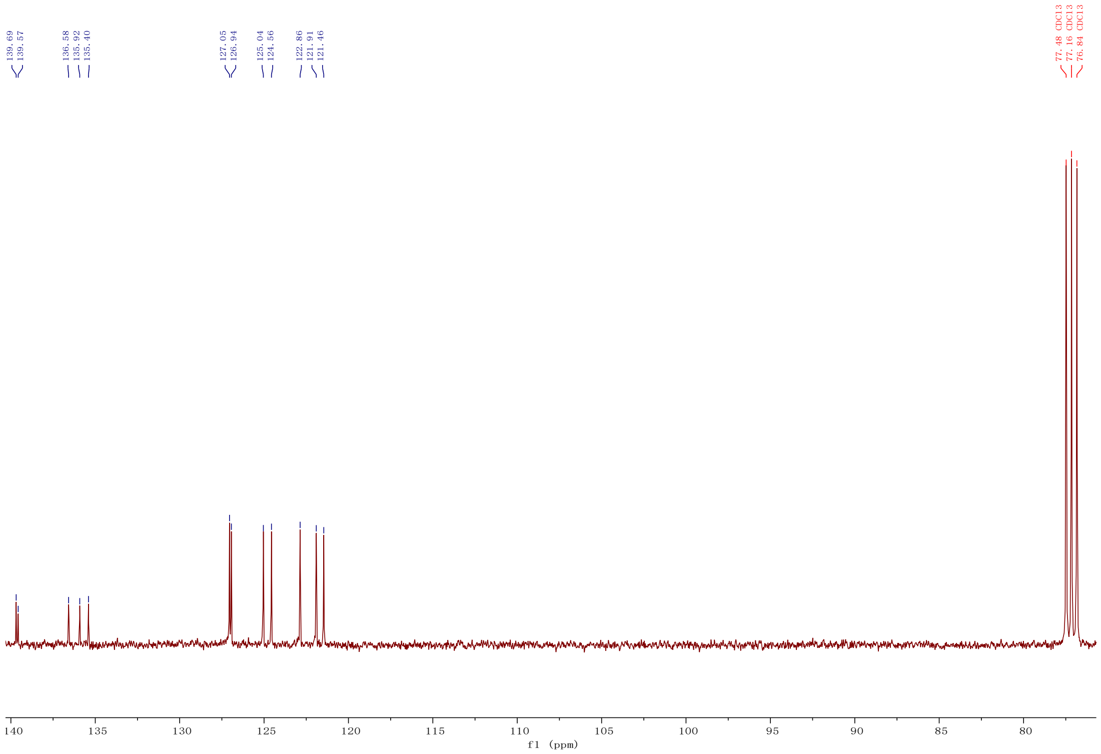

Chart S4 ^{13}C NMR spectrum of DBTDBT in Chloroform-*d*.

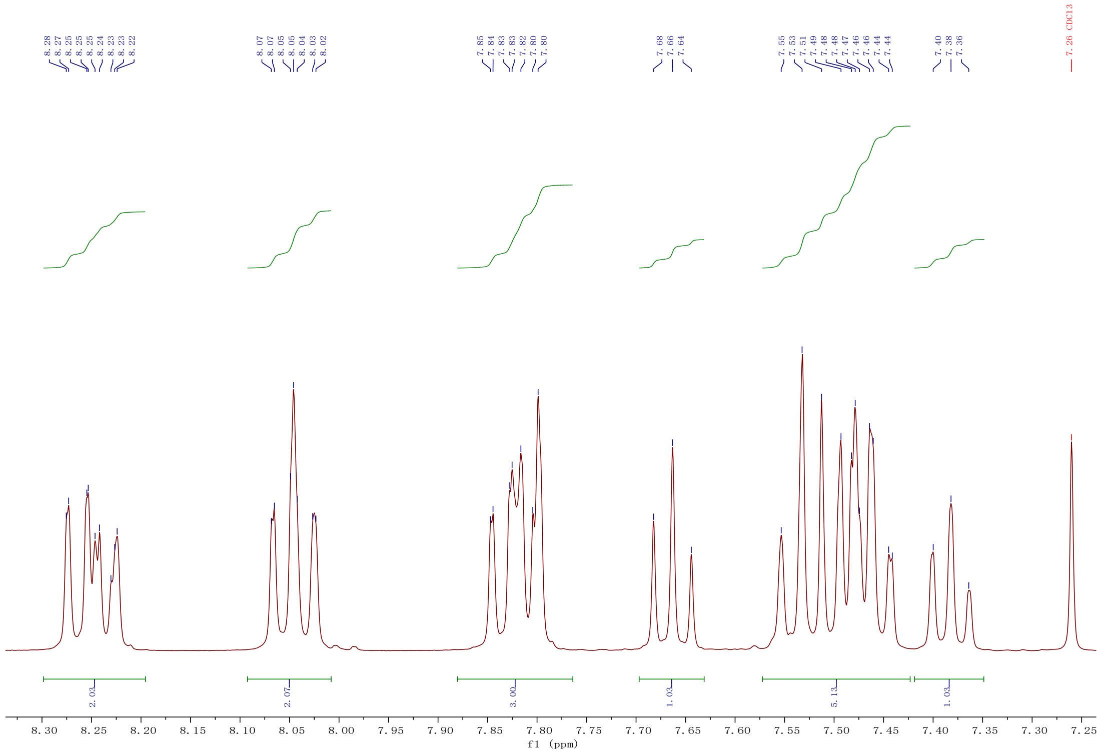

Chart S5 ^{1}H NMR spectrum of DBFDBT in Chloroform-*d*.

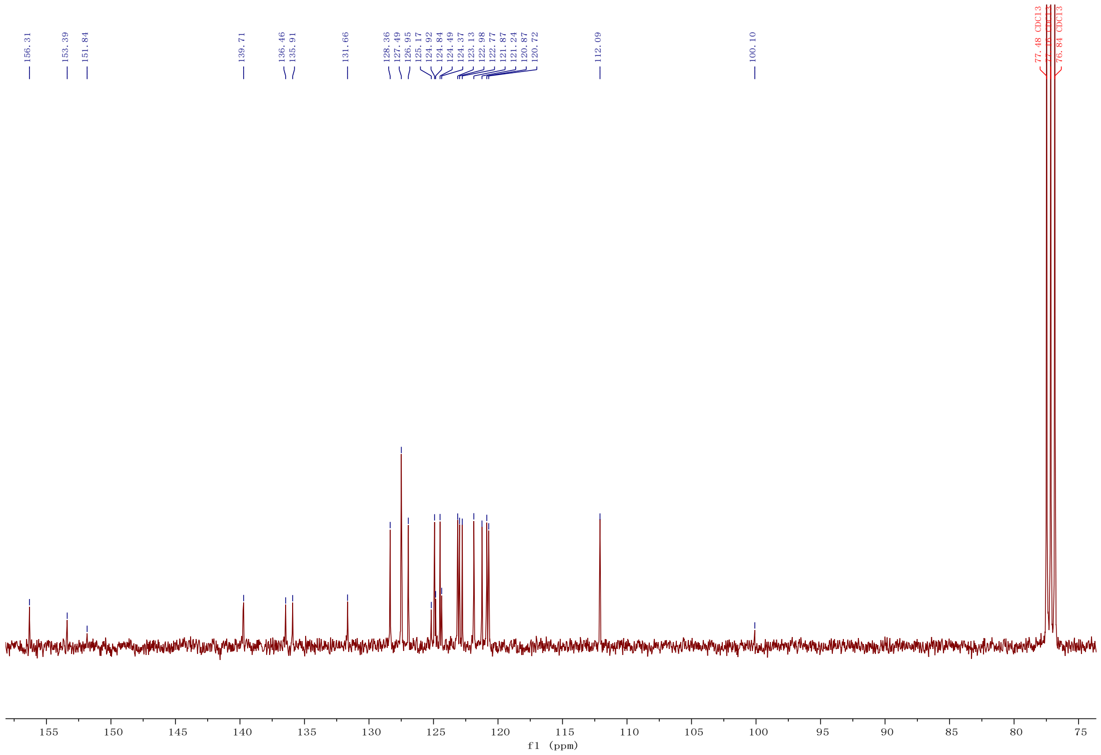

Chart S6 ^{13}C NMR spectrum of DBFDBT in Chloroform-*d*.

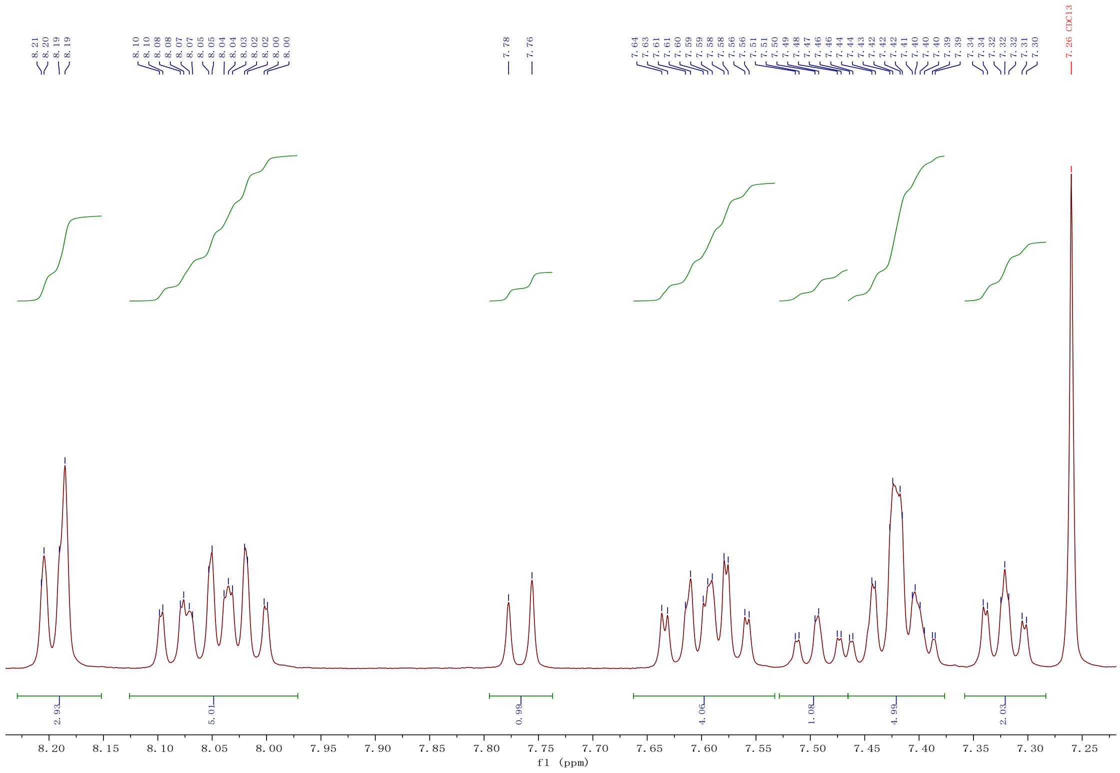

Chart S7 ^{1}H NMR spectrum of DBFDBFCZ in Chloroform-*d*.

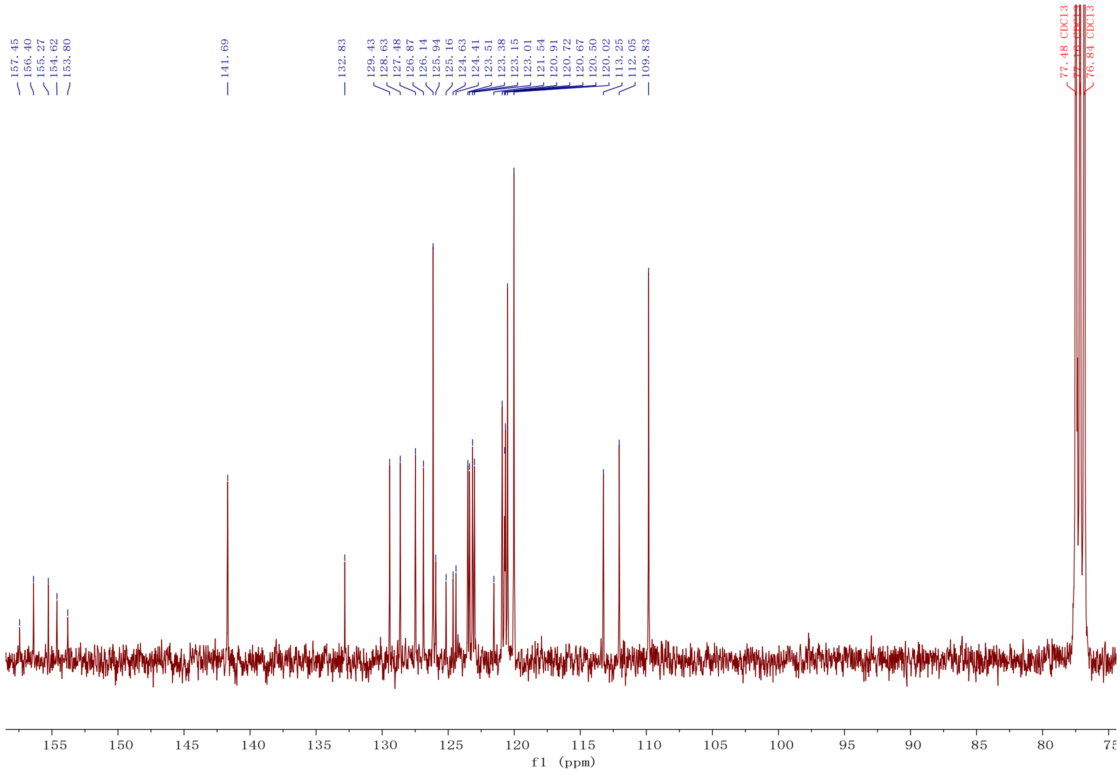

Chart S8 ^{13}C NMR spectrum of DBFDBFCZ in Chloroform-*d*.

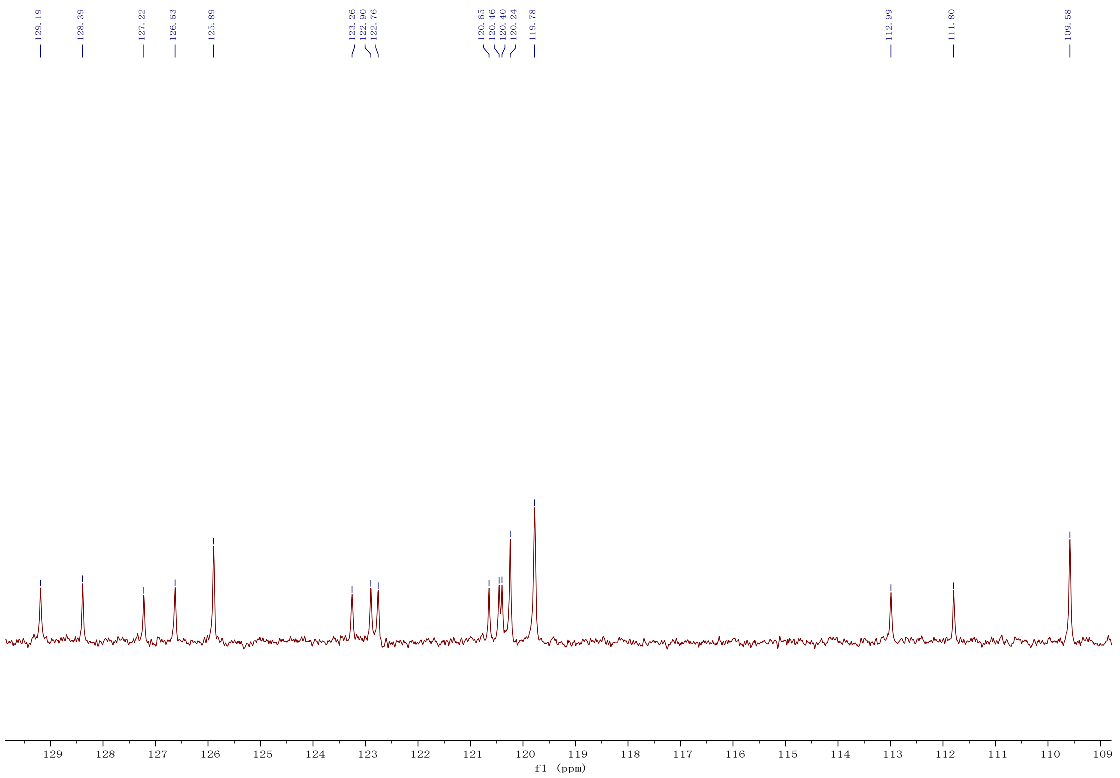

Chart S9 ^{13}C dept135 NMR spectrum of DBFDBFCZ in Chloroform-*d*.

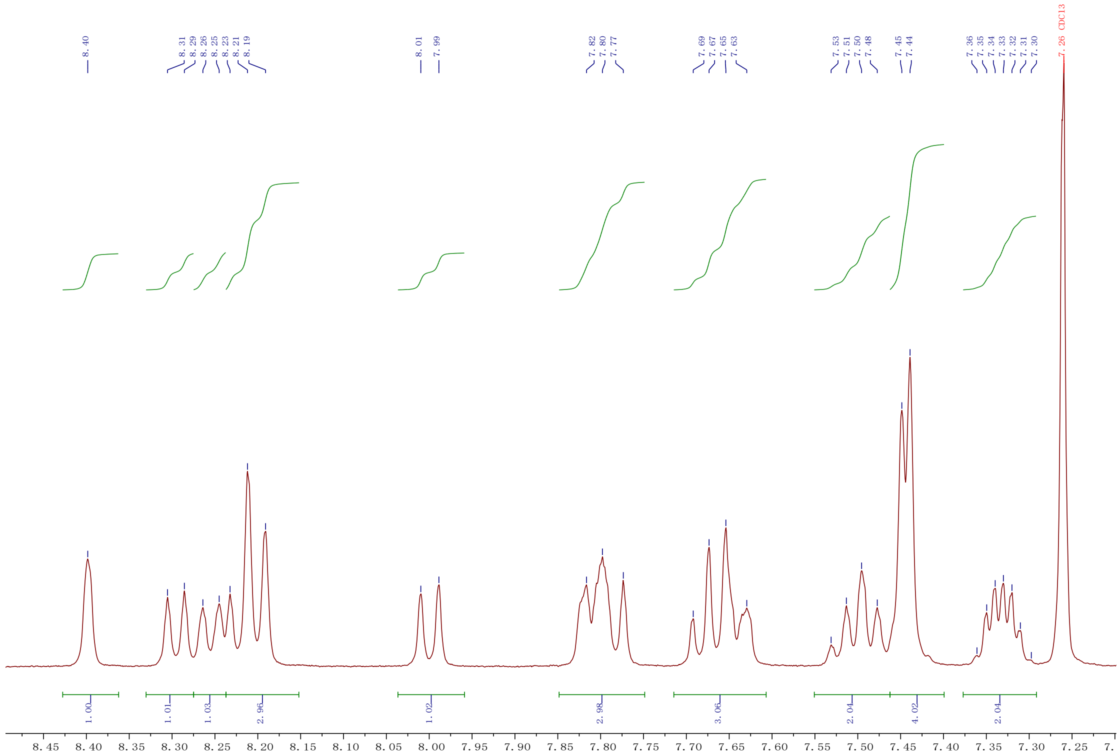

Chart S10 ^{1}H NMR spectrum of DBTDBTCZ in Chloroform-*d*.

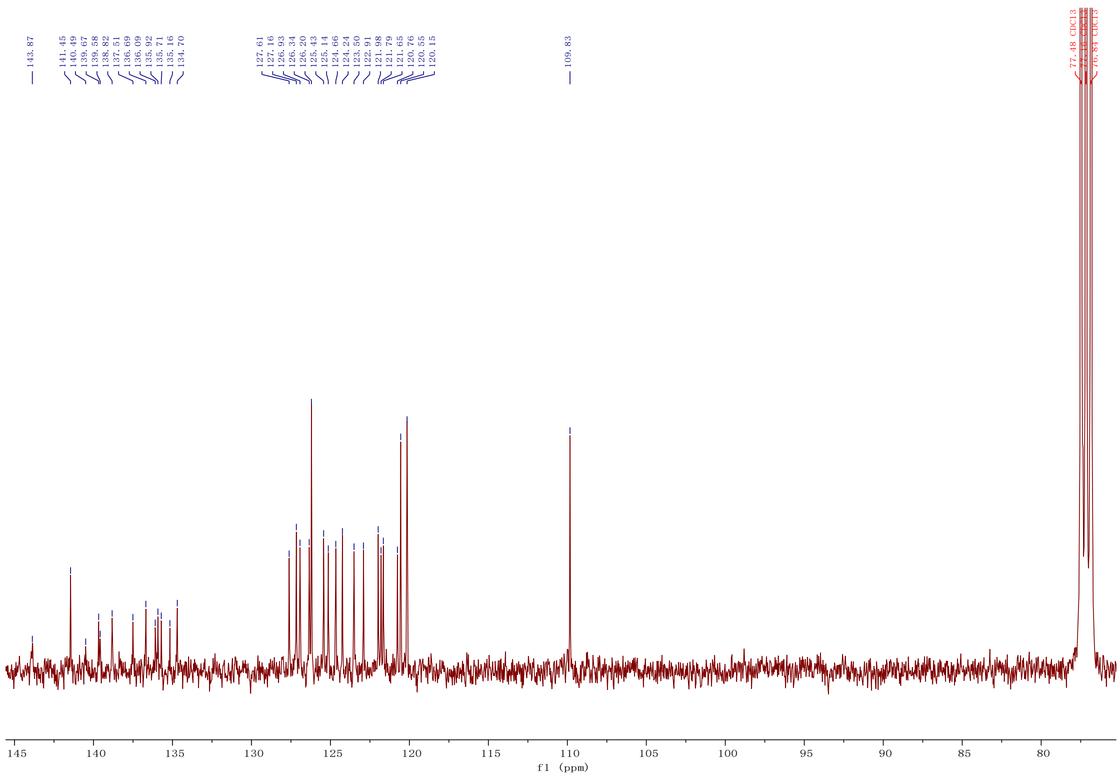

Chart S11 ^{13}C NMR spectrum of DBTDBTCZ in Chloroform-*d*.

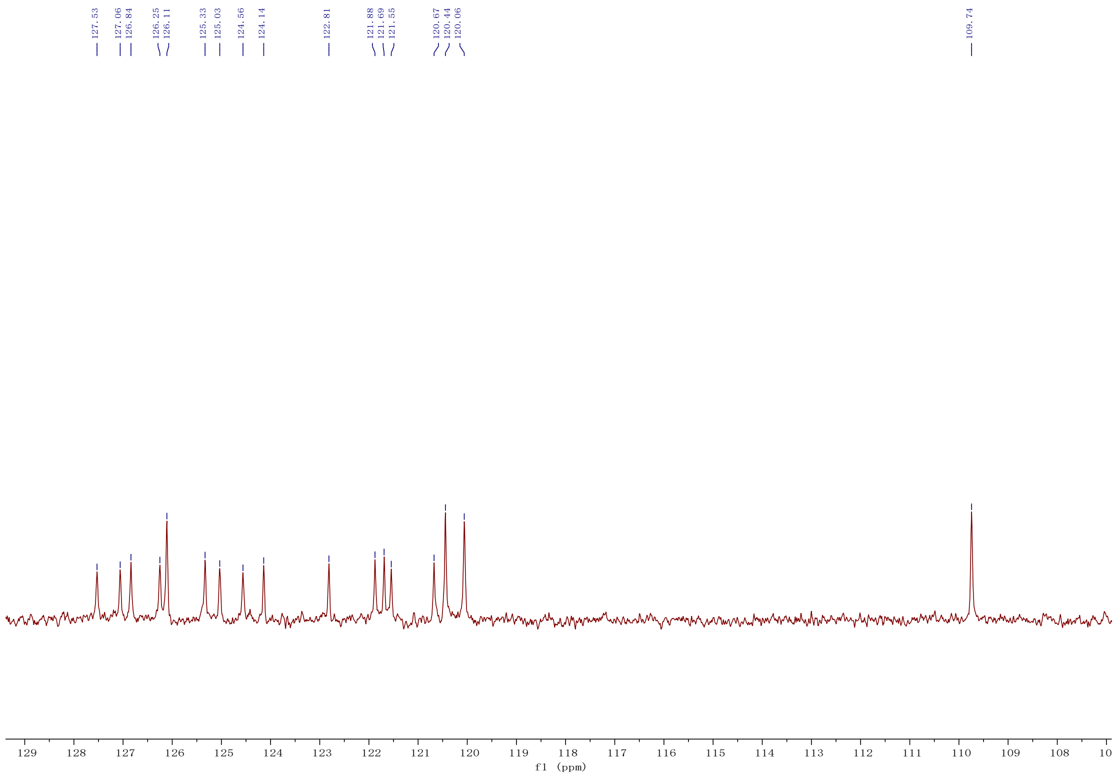

Chart S12 ^{13}C dept135 NMR spectrum of DBTDBTCZ in Chloroform-*d*.

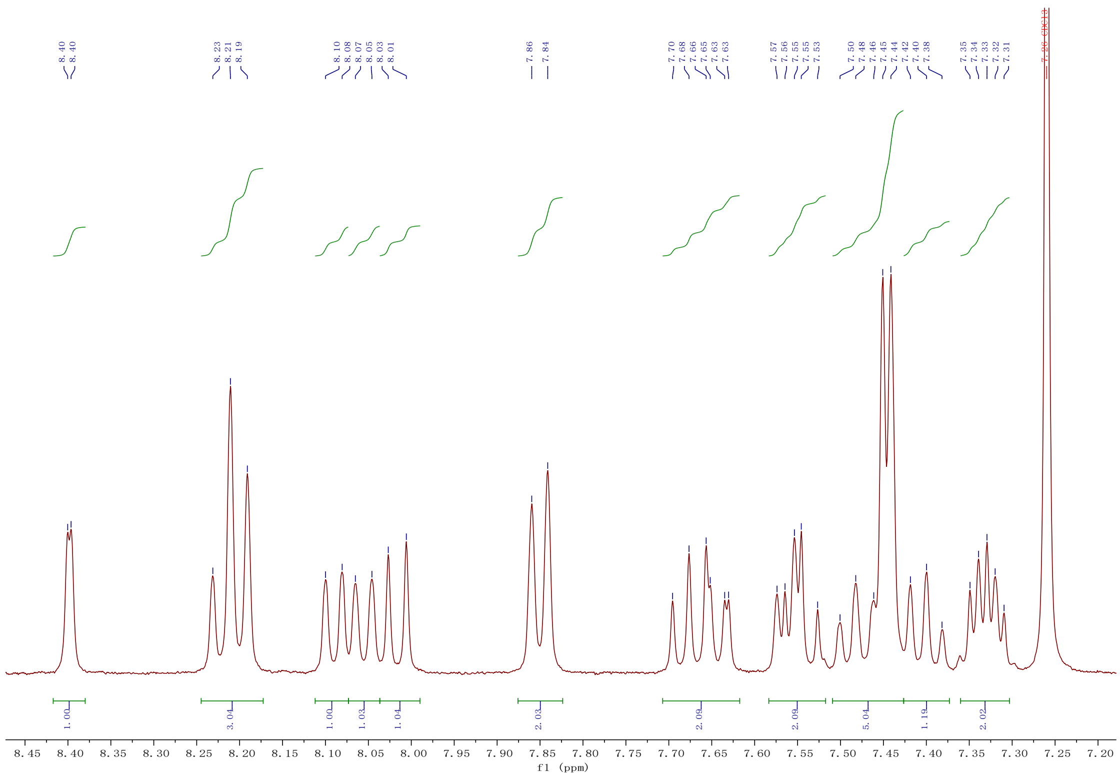

Chart S13 ^{1}H NMR spectrum of DBFDBTCZ in Chloroform-*d*.

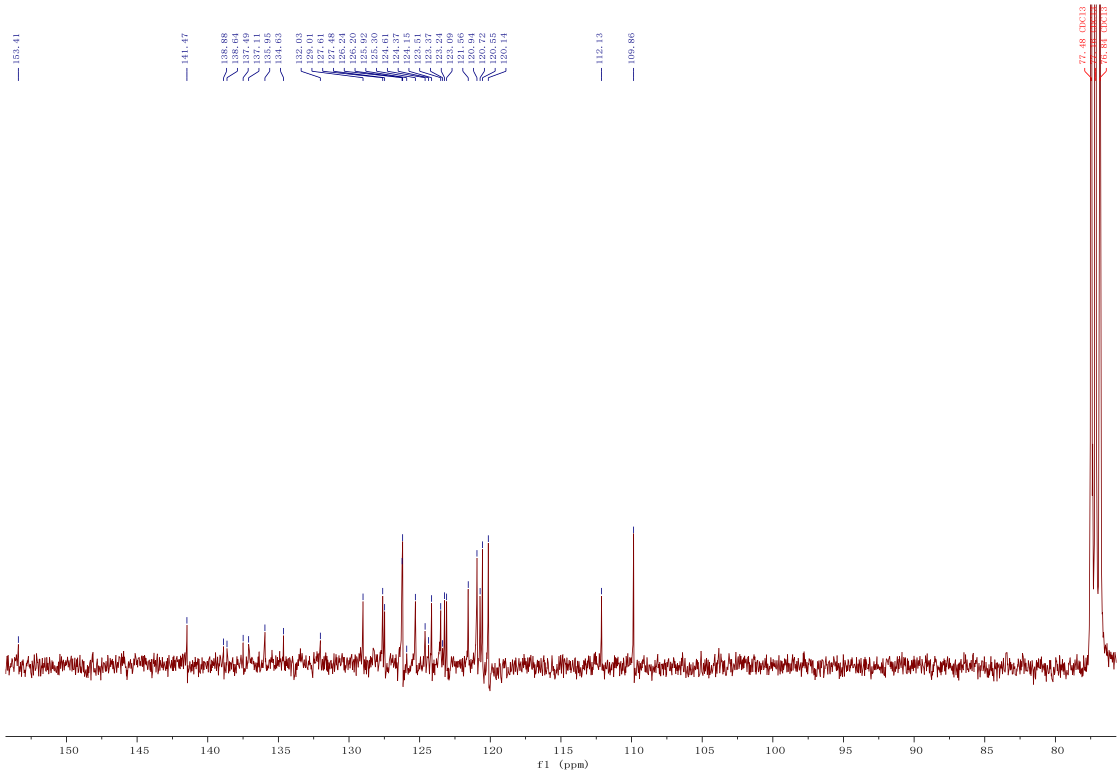

Chart S14 ^{13}C NMR spectrum of DBFDBTCZ in Chloroform-*d*.

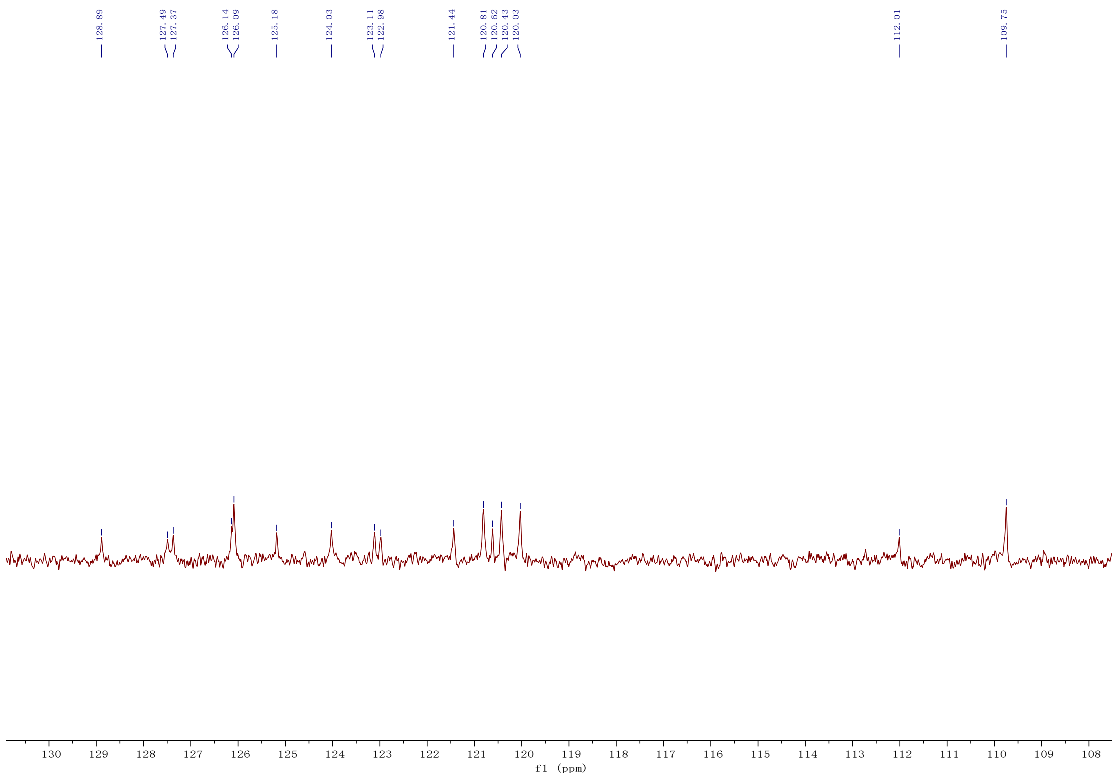

Chart S15 ^{13}C dept135 NMR spectrum of DBFDBTCZ in Chloroform-*d*.

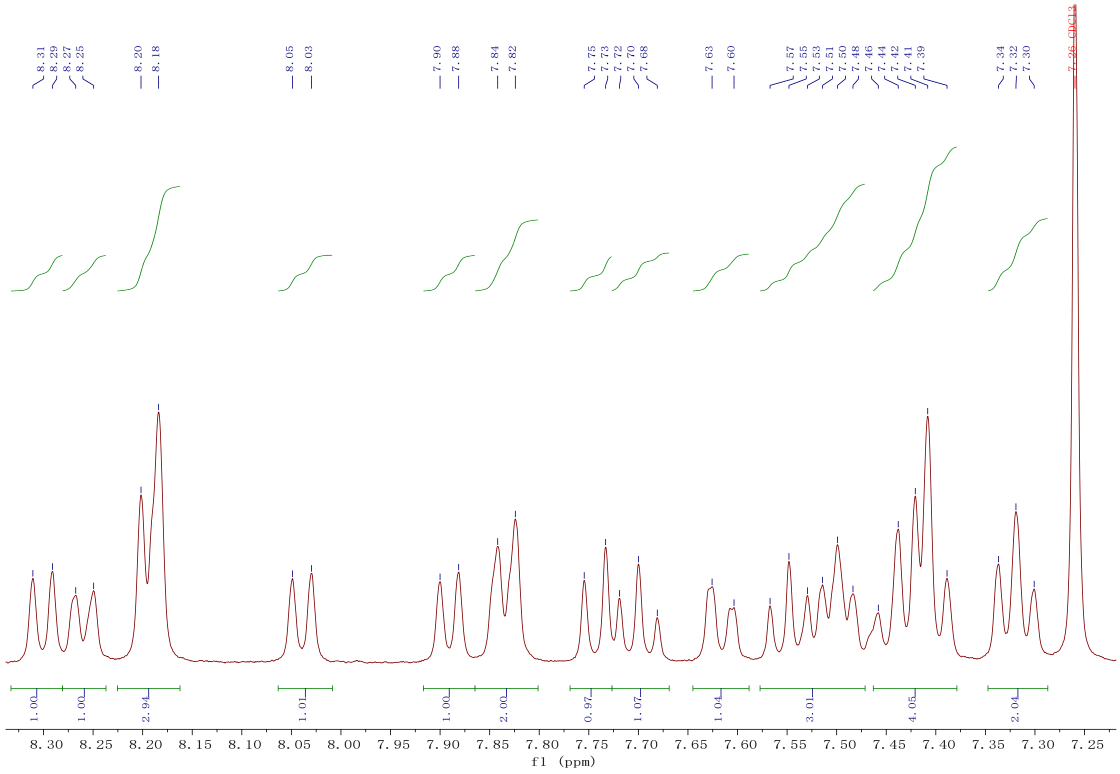

Chart S16 ^{1}H NMR spectrum of DBTDBFCZ in Chloroform-*d*.

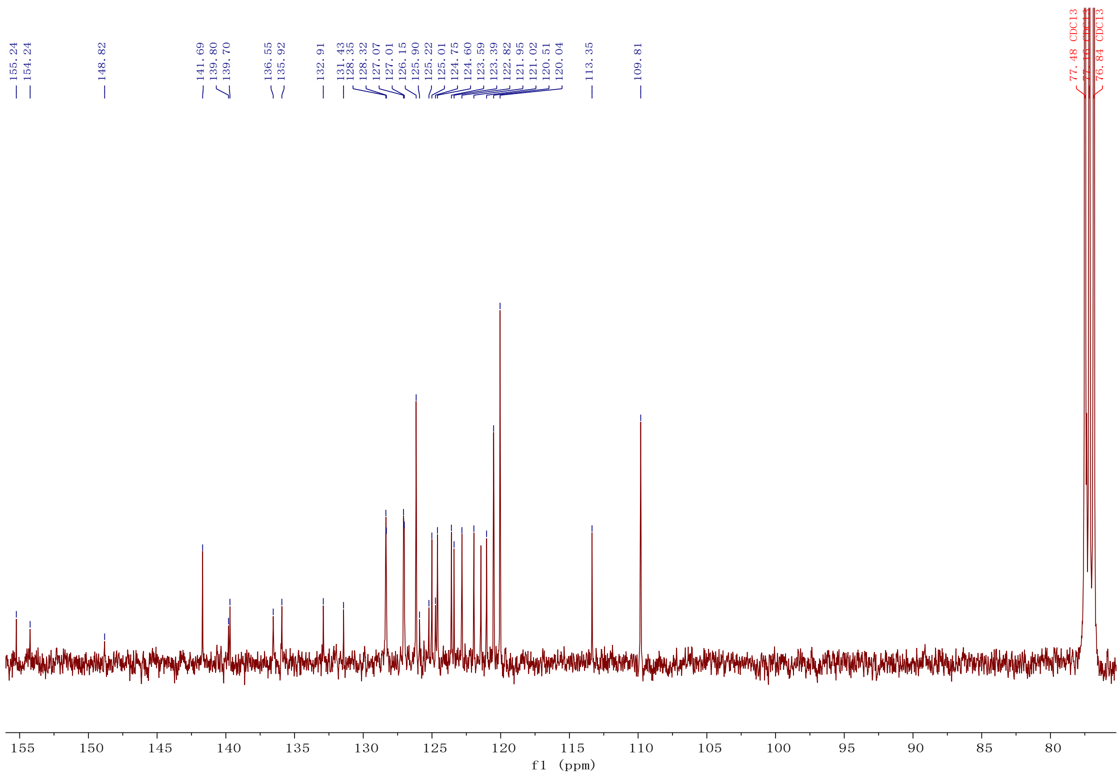

Chart S17 ^{13}C NMR spectrum of DBTDBFCZ in Chloroform-*d*.

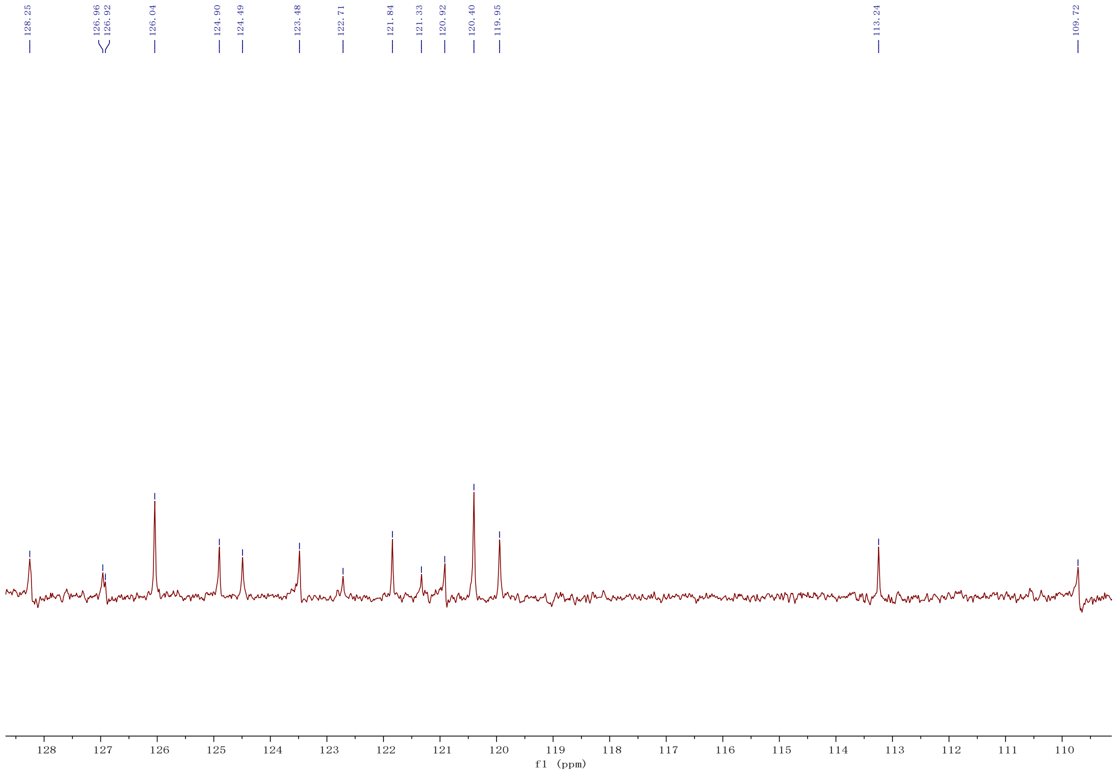

Chart S18 ^{13}C dept135 NMR spectrum of DBTDBFCZ in Chloroform-*d*.

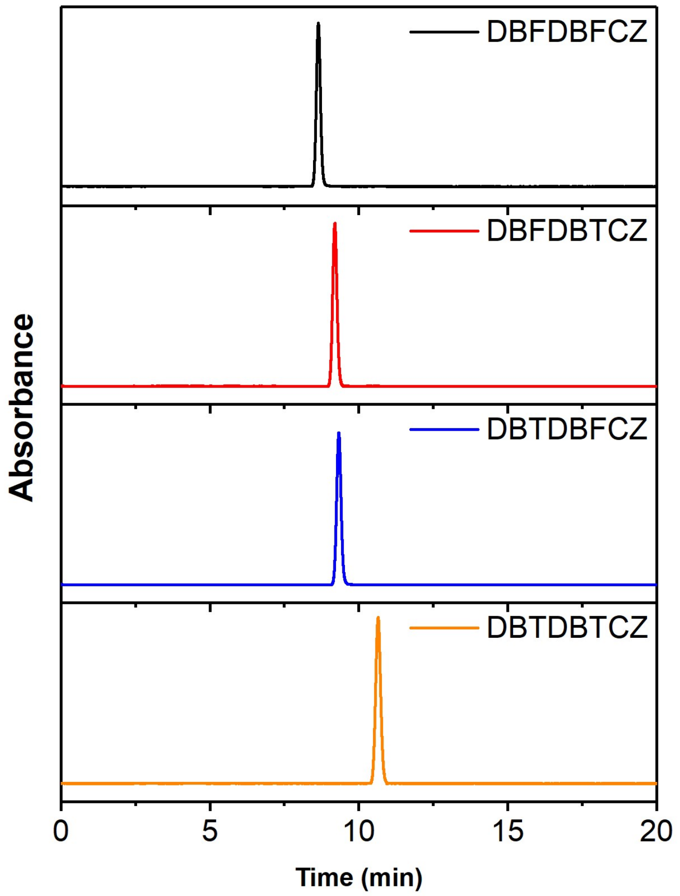

Chart S19 High performance liquid chromatography of DBFDBFCZ, DBFDBTCZ, DBTDBFCZ and DBTDBTCZ (Temperature: 30°C; Mobile phase: 95% acetonitrile and 5% water; Flow rate: 1 mL/min; Column: WondaSil C18 Superb 5 µm 4.6 × 250 mm; Detection signal: 300 nm).

Crystal data and structure refinement

Table S1. Crystal Data and Structure Refinement for DBTDBFCZ.

Empirical formula	C36 H21 N O S
Formula weight	515.60
Temperature	303.7(4) K
Wavelength	1.54184 Å
Crystal system	monoclinic
Space group	P 1 21/n 1
Unit cell dimensions	a = 24.7977(11) Å alpha = 90 deg.
	b = 3.9818(2) Å beta = 97.267(4) deg.
	c = 24.8783(10) Å gamma = 90 deg.
Volume	2436.74(19) $Å^3$
Z	4
Density (calculated)	1.405 Mg/m^3
Absorption coefficient	1.428 mm^{-1}
F(000)	1072.0
Crystal size	0.4 x 0.3 x 0.1 mm^3
Theta range for data collection	2.370 to 75.486
Index ranges	-30<=h<=31 -2<=k<=4, -30<=l<=30
Reflections collected	12504
Independent reflections	4824 [R(int) = 0.0744]
Absorption correction	multi-scan
Max. and min. transmission	1.00000 and 0.09779
Refinement method	Least-squares minimization
Data / restraints / parameters	4824 / 0 /352
Goodness-of-fit on F2	0.923
Final R indices [I>2sigma(I)]	R1 = 0.0537, wR2 = 0.1218
R indices (all data)	R1 = 0.1137, wR2 = 0.1524
Largest diff. peak and hole	0.225 and -0.431 e.$Å^{-3}$

Table S2. Crystal Data and Structure Refinement for DBTDBTCZ.

Empirical formula	C36 H21 N S2
Formula weight	531.66
Temperature	99.99(10) K
Wavelength	1.54184 Å
Crystal system	orthorhombic
Space group	P c a 21
Unit cell dimensions	a = 19.1082(12) Å alpha = 90 deg.
	b = 3.9400(2) Å beta = 90 deg.
	c = 32.2145(17) Å gamma = 90 deg.
Volume	2425.3(2) $Å^3$
Z	4
Density (calculated)	1.456 Mg/m^3
Absorption coefficient	2.204 mm^{-1}
F(000)	1104.0
Crystal size	0.5 x 0.3 x 0.2 mm^3
Theta range for data collection	2.743 to 76.318
Index ranges	-23<=h<=23, -4<=k<=3, -37<=l<=39
Reflections collected	6853
Independent reflections	3482 [R(int) = 0.0768]
Absorption correction	Multi-scan
Max. and min. transmission	1.00000 and 0.42293
Refinement method	Least-squares minimization
Data / restraints / parameters	3482 / 1 /353
Goodness-of-fit on F2	1.133
Final R indices [I>2sigma(I)]	R1 = 0.0792, wR2 = 0.2408
R indices (all data)	R1 = 0.0978, wR2 = 0.2730
Largest diff. peak and hole	0.652 and -0.808 e.$Å^{-3}$

Calculated HOMOs and LUMOs

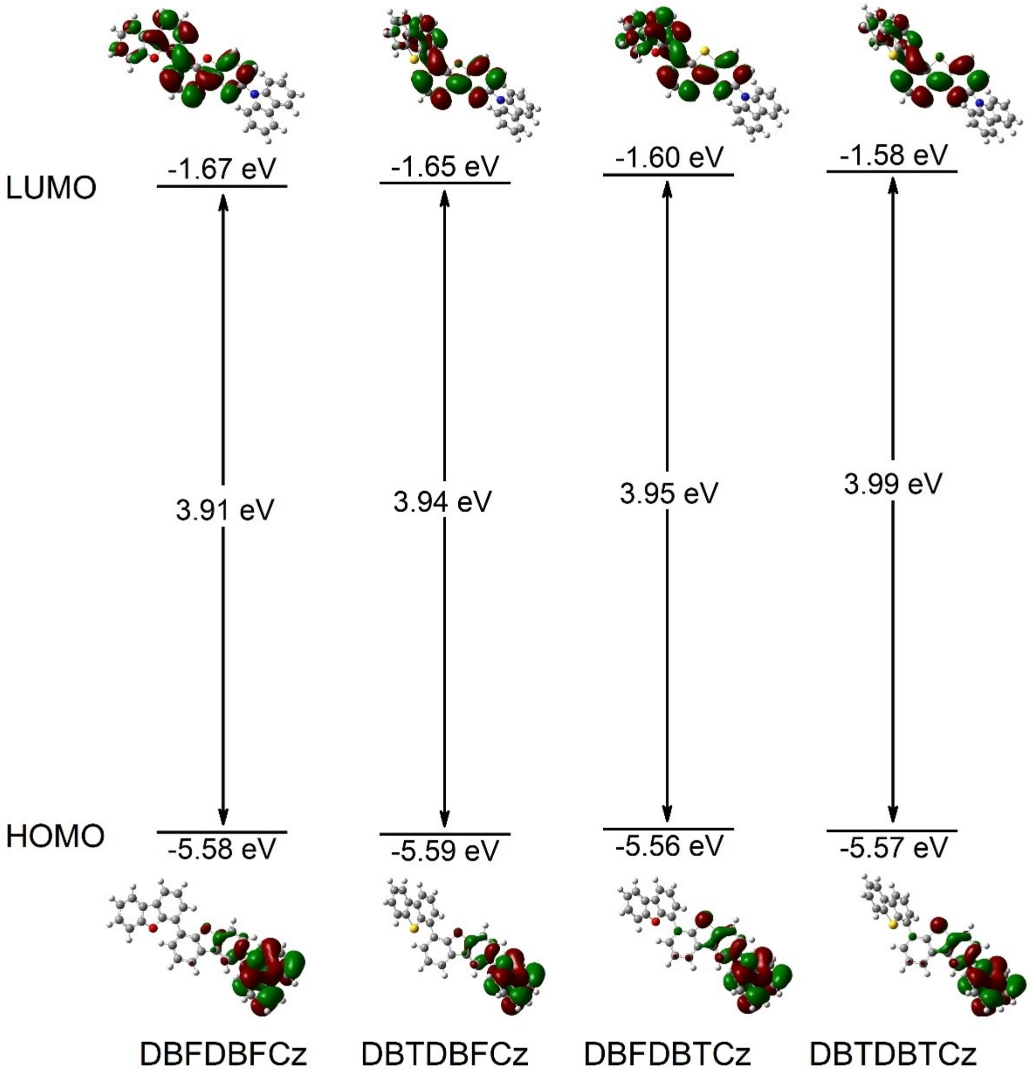

Fig. S1 The frontier orbits of molecules calculated at b3lyp/6-311g(d,p) level.

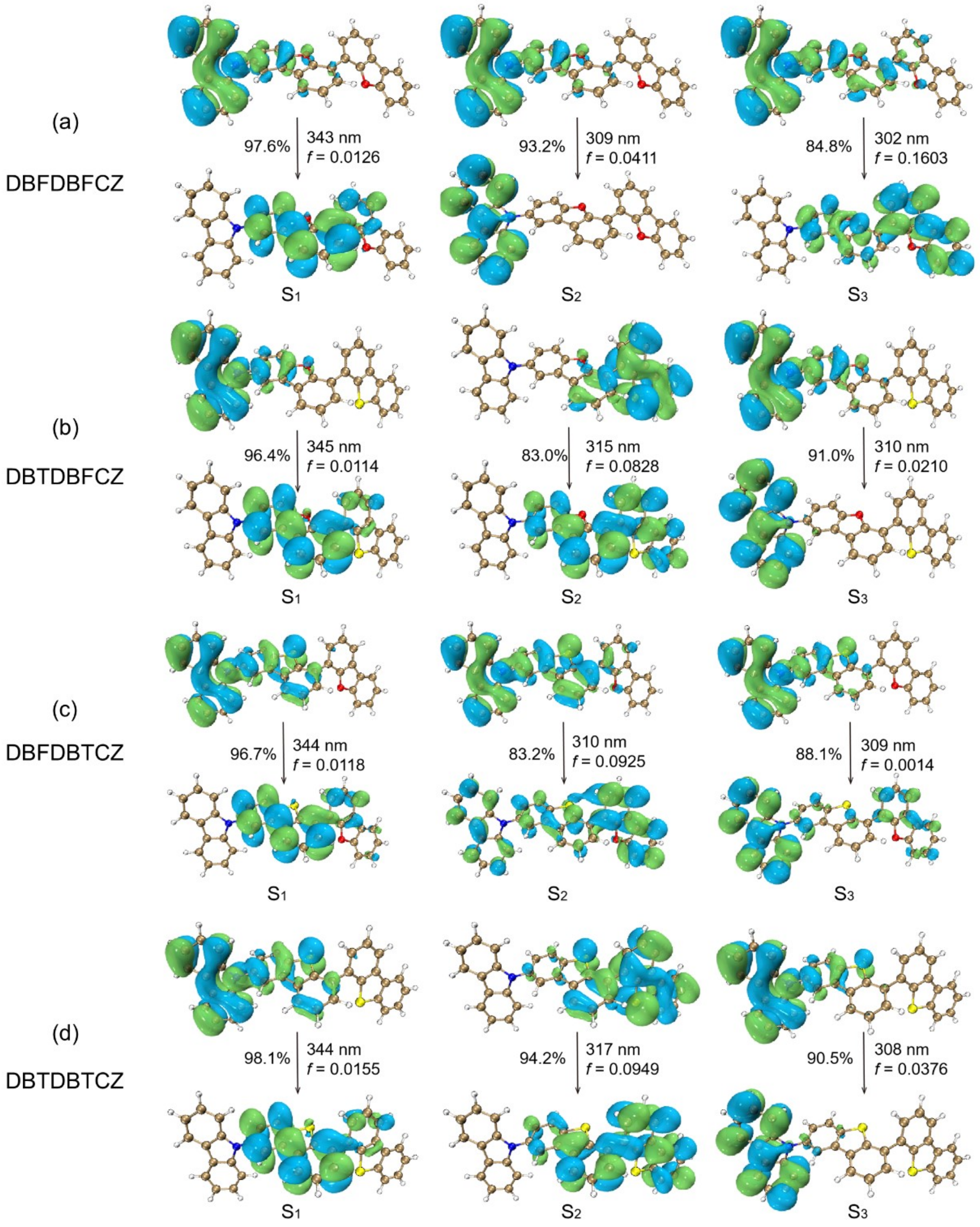

Fig. S2 The natural transition orbitals of frontier excited singlet states and oscillator strength (*f*) for (a) DBFDBFCZ, (b) DBTDBFCZ, (c) DBFDBTCZ and (d) DBTDBTCZ in the monomeric state at (TD)B3LYP/def2-SVP level.

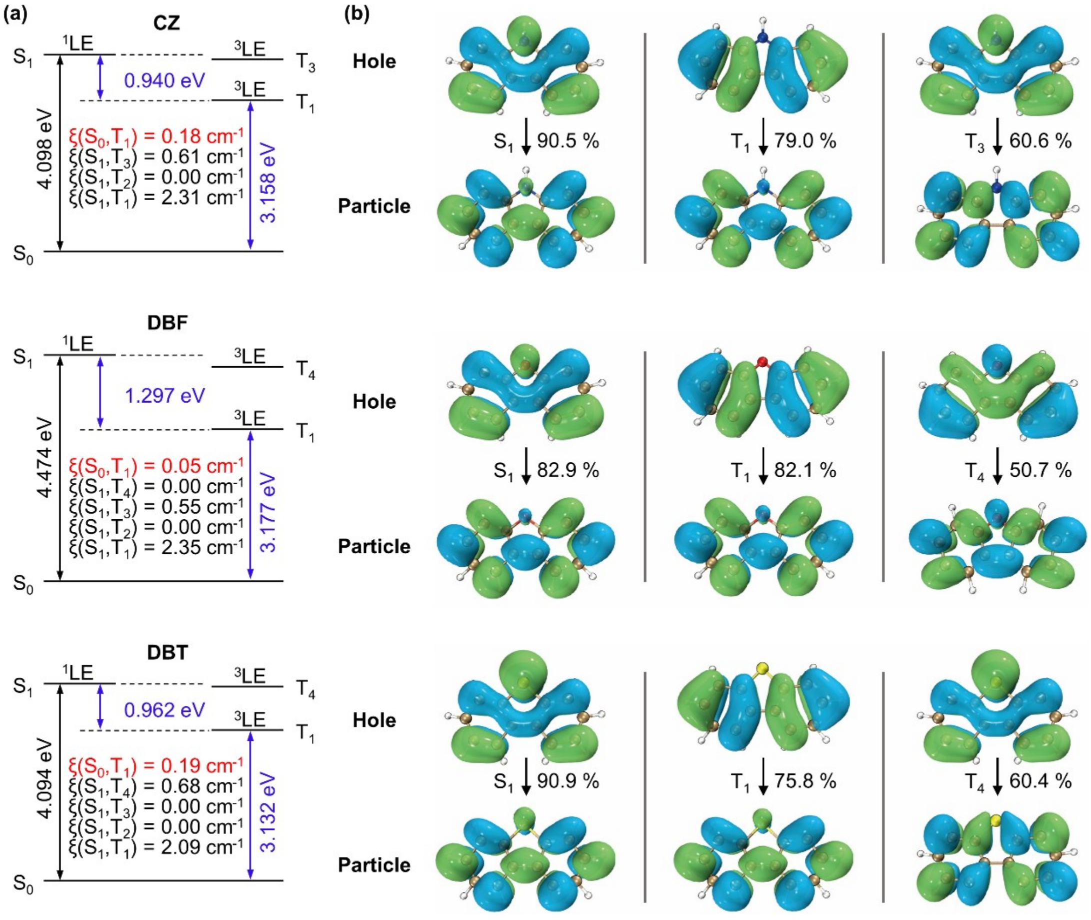

Fig. S3 (a) The energy-level diagrams, molecular orbital characters, SOC coefficients (ξ) and (b) the natural transition orbitals of frontier excited singlet states and excited triplet states for Cz, DBF and DBT in the monomeric state at (TD)B3LYP/def2-SVP level.

Supplementary photophysical data

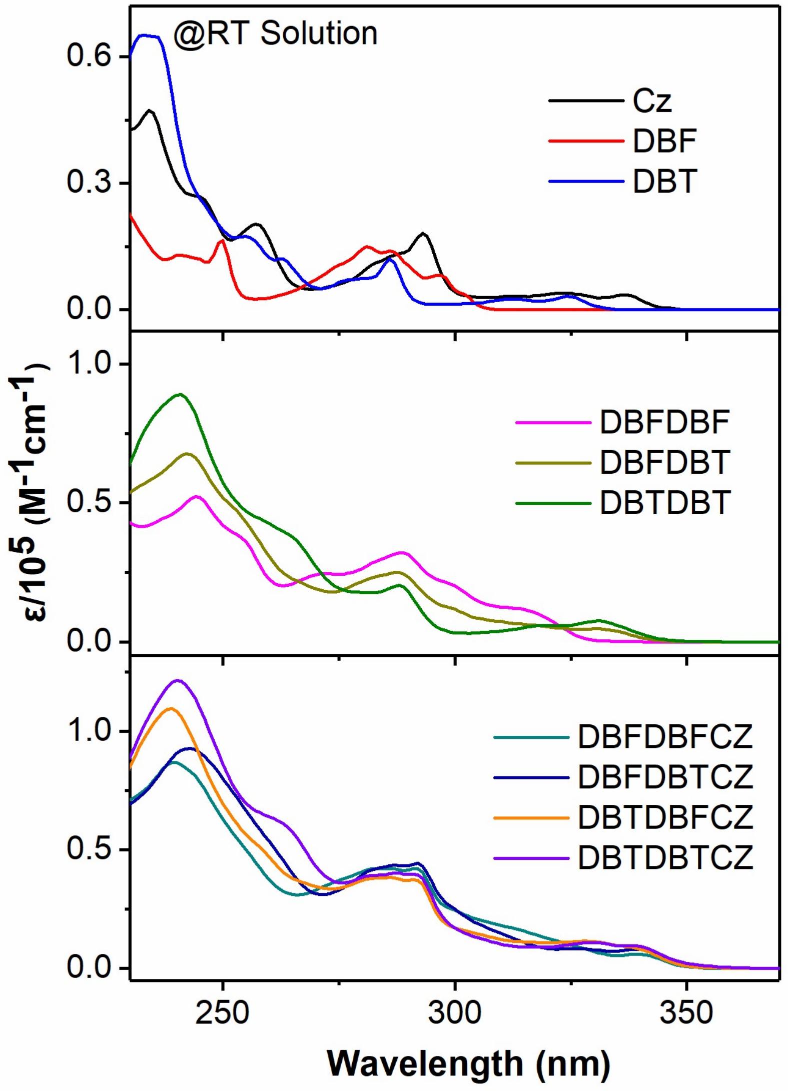

Fig. S4 UV-Visible absorption spectra at room temperature of CZ, DBF, DBT; DBFDBF, DBFDBT, DBTDBT; DBFDBFCZ, DBFDBTCZ, DBTDBFCZ and DBTDBTCZ in 2-methyltetrahydrofuran (10^{-5} M).

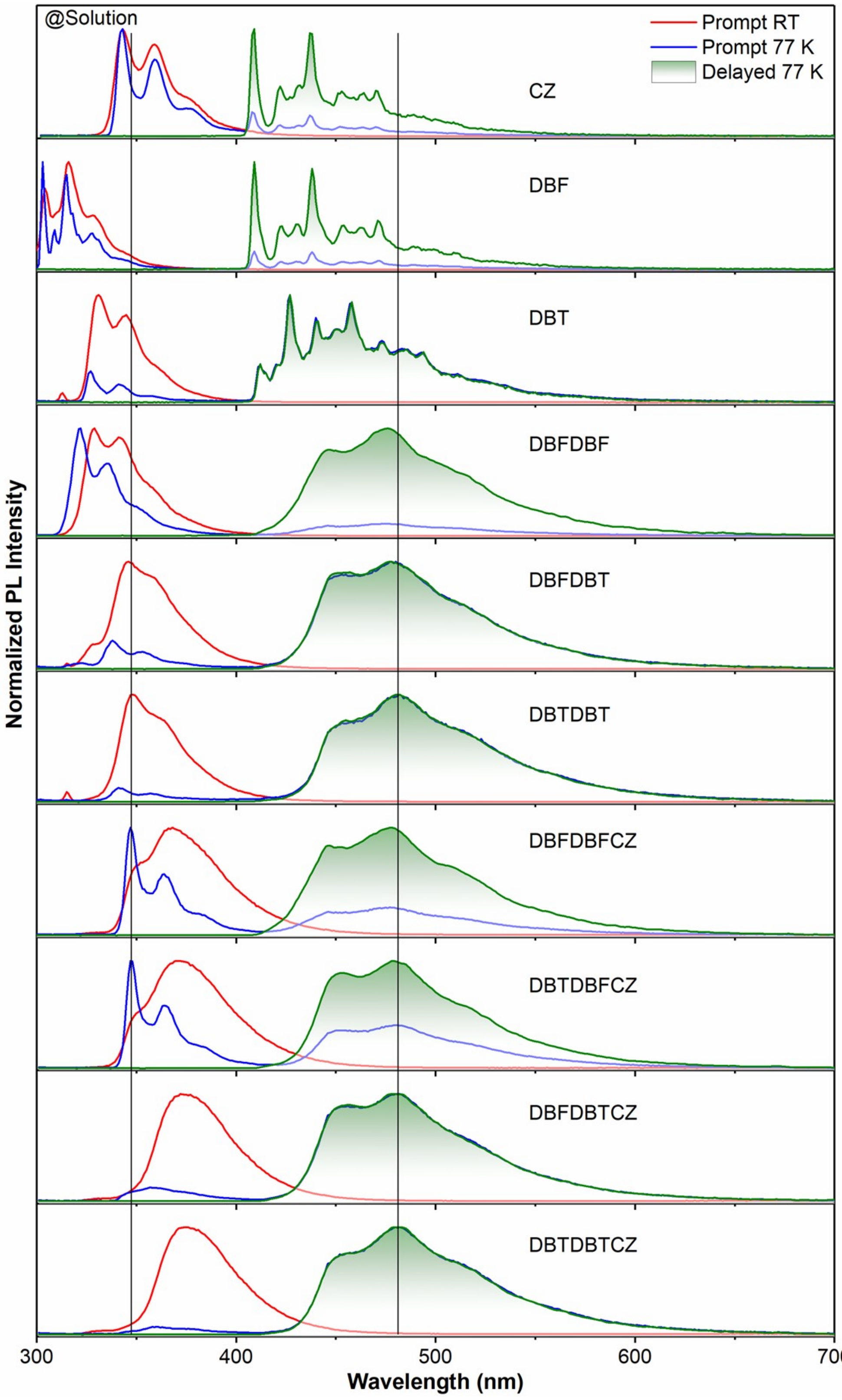

Fig. S5 Normalized photoluminescence spectra at room temperature and 77 K, and normalized delayed (1 ms) spectra at 77 K of CZ, DBF, DBT, DBFDBF, DBFDBT, DBTDBT, DBFDBFCZ, DBTDBFCZ, DBFDBTCZ and DBTDBTCZ in 2-methyltetrahydrofuran (10^{-5} M).

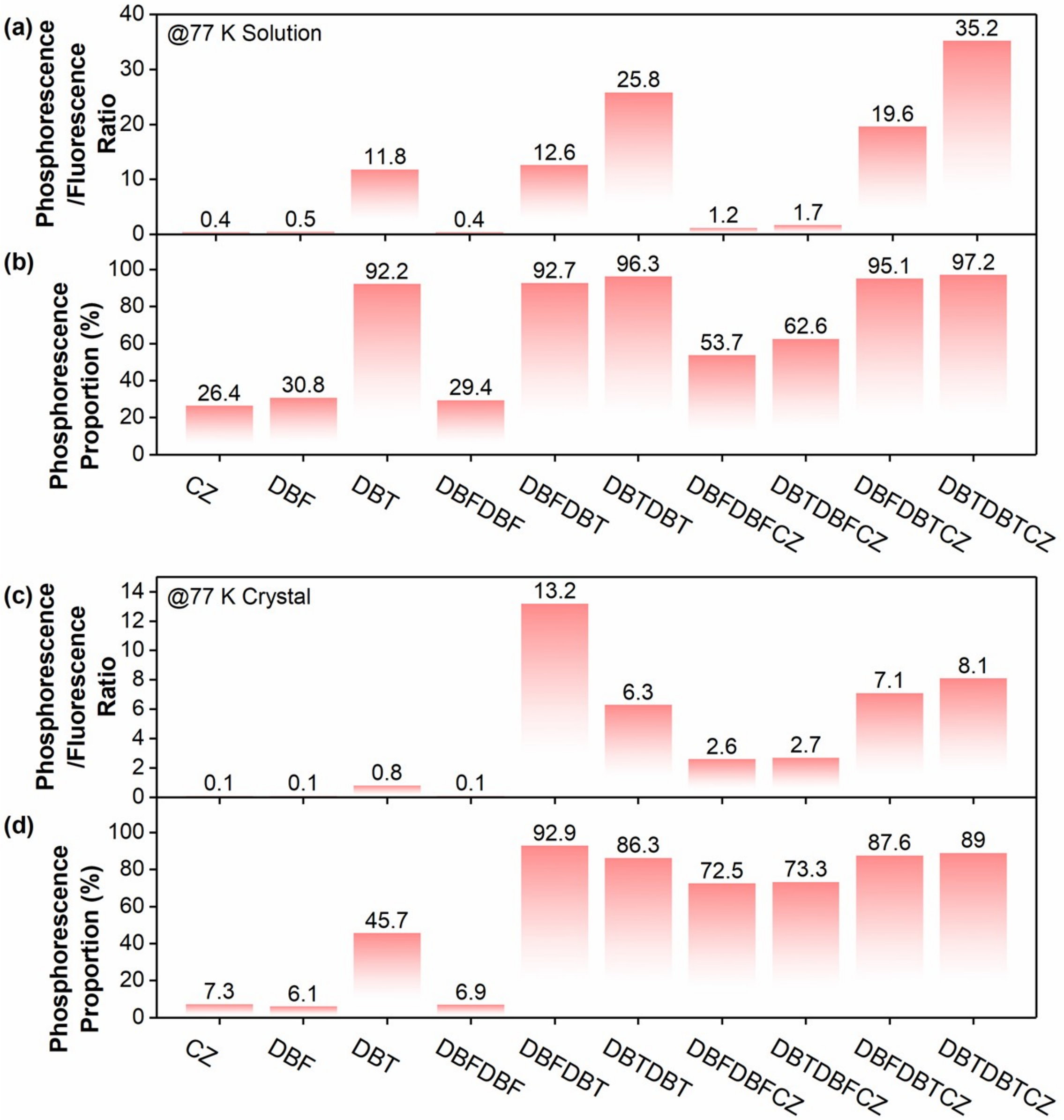

Fig. S6 (a) The ratio of phosphorescence to fluorescence, (b) phosphorescence proportion of CZ, DBF, DBT, DBFDBF, DBFDBT, DBTDBT, DBFDBFCZ, DBTDBFCZ, DBFDBTCZ and DBTDBTCZ in 2-methyltetrahydrofuran at 77 K (10^{-5} M). (c) The ratio of phosphorescence to fluorescence, (d) phosphorescence proportion of CZ, DBF, DBT, DBFDBF, DBFDBT, DBTDBT, DBFDBFCZ, DBFDBTCZ, DBTDBFCZ and DBTDBTCZ crystal at 77 K.

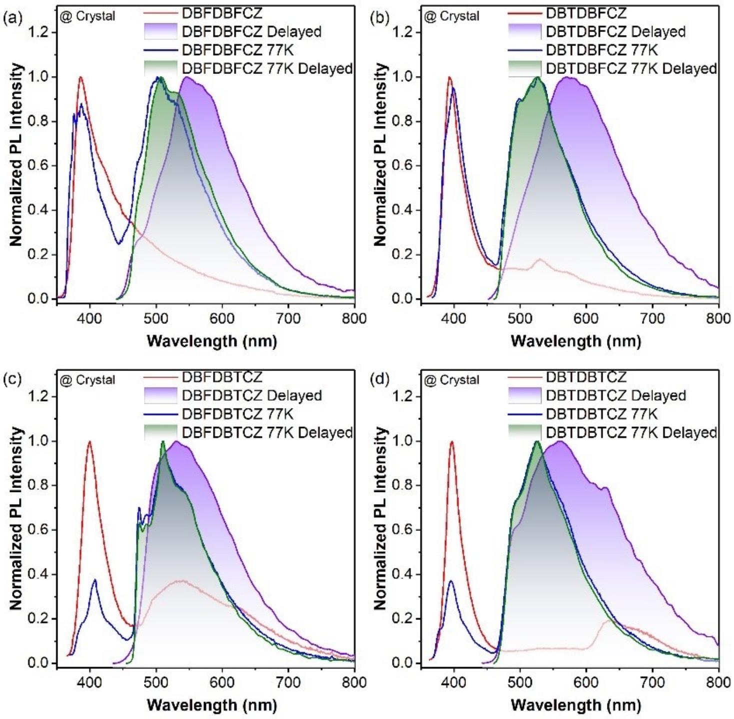

Fig. S7 (a) Normalized photoluminescence spectra at room temperature and 77 K, and normalized delayed (1 ms) spectra at room temperature and 77 K of (a) DBFDBFCZ, (b) DBTDBFCZ, (c) DBFDBTCZ and (d) DBTDBTCZ crystal.

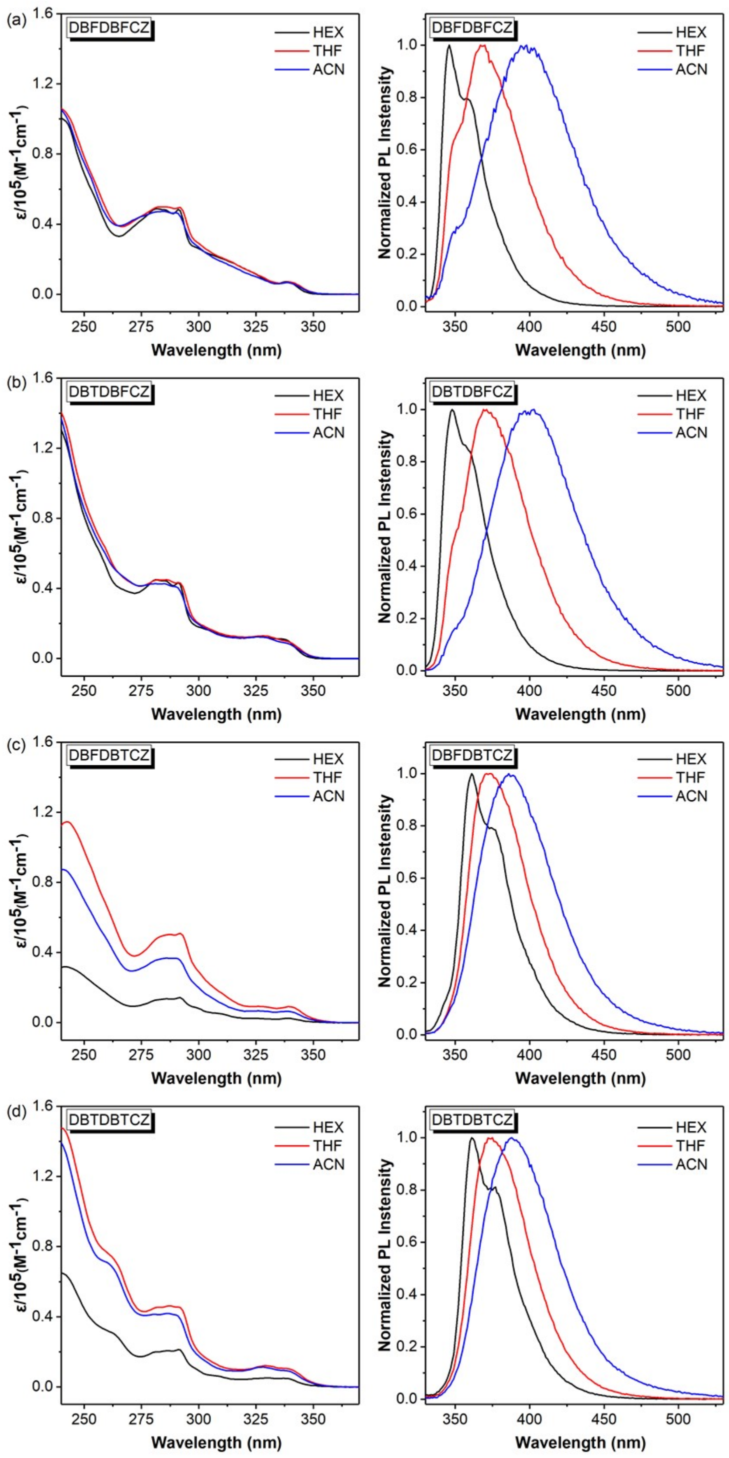

Fig. S8 (a) UV-Visible absorption spectra, and (b) normalized photoluminescence spectra at room temperature of DBFDBFCZ, DBTDBFCZ, DBFDBTCZ and DBTDBTCZ in n-hexane (HEX), tetrahydrofuran (THF) and acetonitrile (ACN) (10^{-5} M).

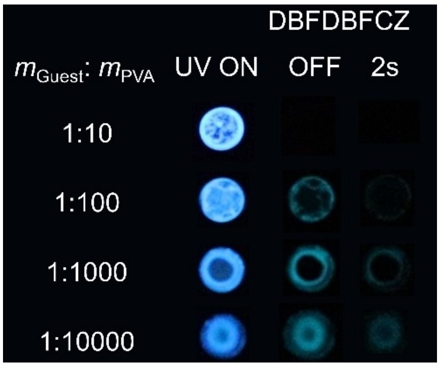

Fig. S9 Photographs of treated films with different concentrations of doping DBFDBFCZ taken before and after removal of the UV excitation source of 254 nm under ambient conditions.

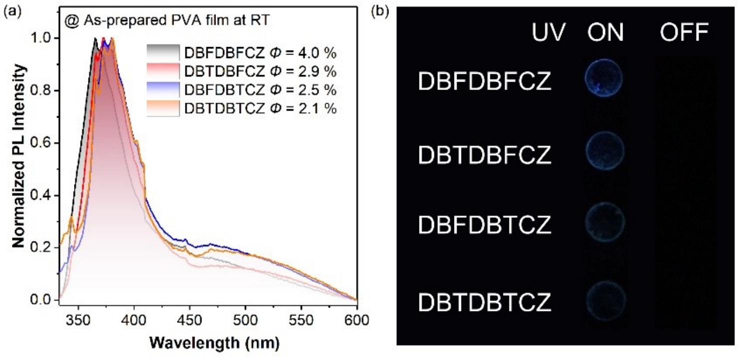

Fig. S10 (a) Normalized photoluminescence spectra at room temperature of as-prepared PVA films with doped ternary π-conjugated molecules under 292 nm excitation. (b) Photographs of as-prepared films taken before and after removal of the UV excitation source of 254 nm under ambient conditions.

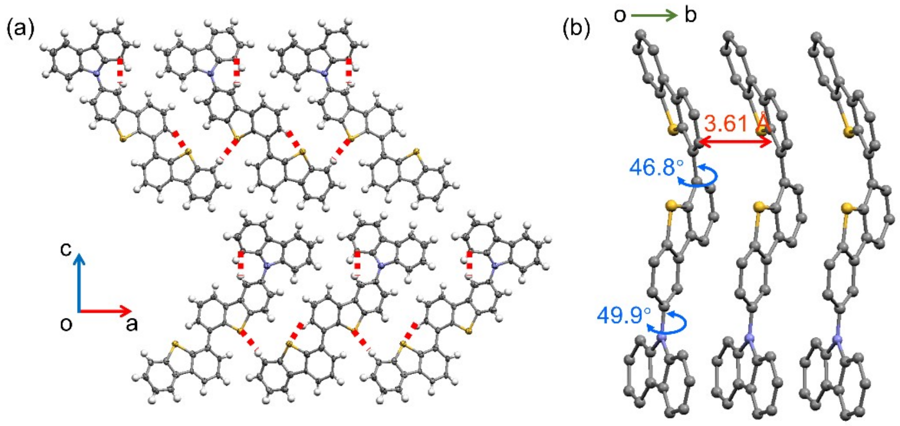

Fig. S11 (a) Crystal arrangement and (b) π-π packing of DBTDBTCZ.

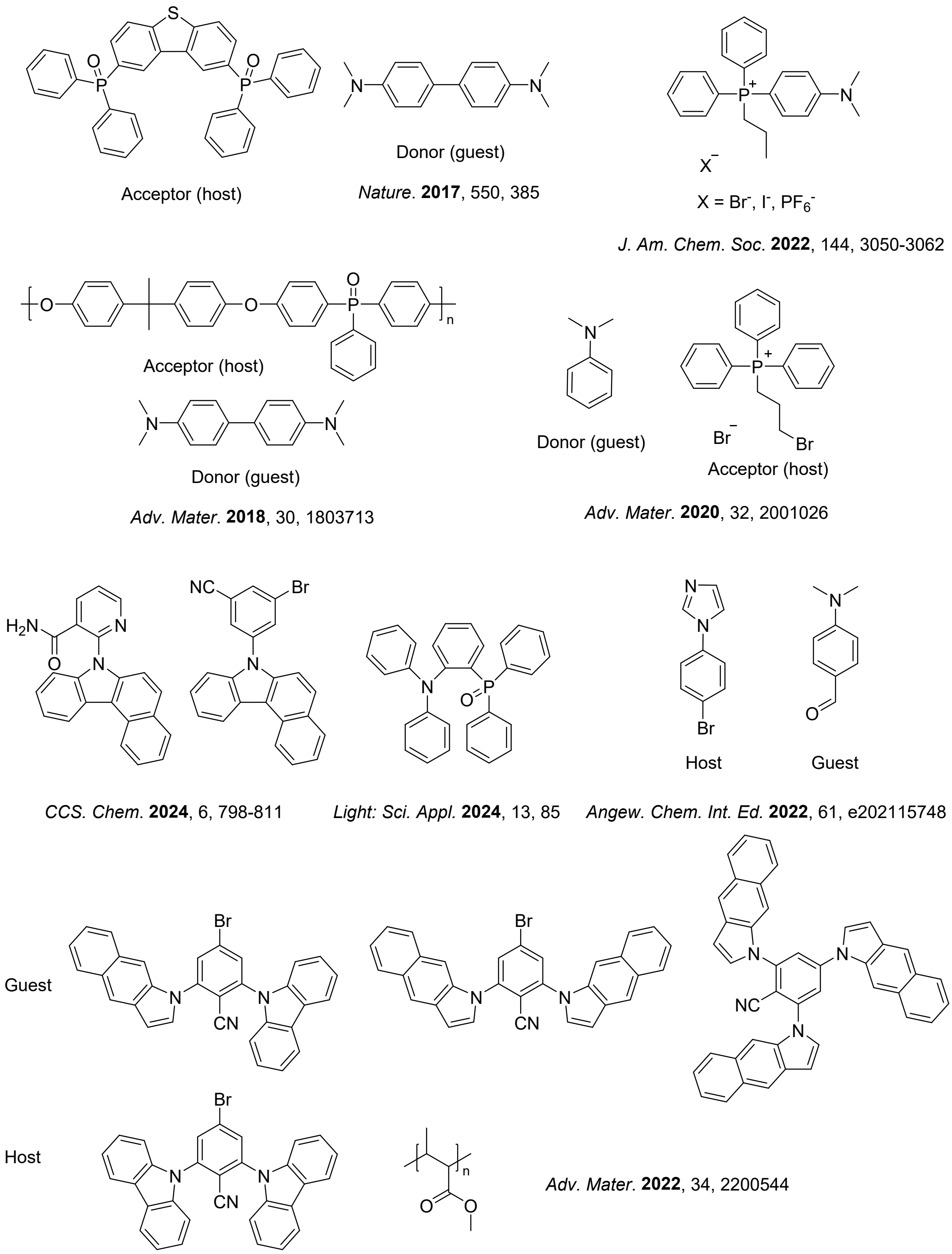

Fig. S12 Reported systems with charge recombination process driven triplet formation[1-8].

Table S3. Photophysical properties of compounds measured at room temperature and 77 K.

Name	State	Emission lifetime	Quantum yield
CZ	Solution (In 2-MeTHF, 1×10^{-5} M)	408 nm (4466.1 ms, 77 K)<br>437 nm (4451.4 ms, 77 K)	51.6 %
	Crystal	365 nm (13.1 ns)<br>356 nm (6.3 ns, 77 K)<br>563 nm (70.3 ms)<br>483 nm (1580.8 ms, 77 K)	46.3 %
DBF	Solution (In 2-MeTHF, 1×10^{-5} M)	409 nm (3075.1 ms, 77 K)<br>438 nm (3074.5 ms, 77 K)	44.4 %
	Crystal	338 nm (6.7 ns)<br>338nm (5.1 ns, 77 K)<br>520 nm (111.7 ms)<br>513 nm (1713.7 ms, 77 K)	35.5 %
DBT	Solution (In 2-MeTHF, 1×10^{-5} M)	427 nm (814.9 ms, 77 K)<br>458 nm (809.0 ms, 77 K)	4.5 %
	Crystal	358 nm (1.0 ns)<br>351 nm (3.5 ns, 77 K)<br>557 nm (195.8 ms)<br>502 nm (1796.7 ms, 77 K)	5.8 %
DBFDBF	Solution (In 2-MeTHF, 1×10^{-5} M)	329 nm (2.4 ns)<br>350 nm (2.3 ns, 77 K)<br>446 nm (1568.8 ms, 77 K)<br>475 nm (1615.8 ms, 77 K)	76.5 %

	Crystal	357 nm (3.1 ns)<br>354 nm (2.7 ns, 77 K)<br>613 nm (11.7 ms)<br>518 nm (1216.9 ms, 77 K)	23.8 %
DBFDBT	Solution (In 2-MeTHF, 1×10^{-5} M)	346 nm (1.1 ns)<br>353 nm (0.5 ns, 77 K)<br>453 nm (442.8 ms, 77 K)<br>477 nm (435.6 ms, 77 K)	6.4 %
	Crystal	380nm (0.4 ns)<br>383 nm (0.3 ns, 77 K)<br>610 nm (16.4 ms)<br>534 nm (618.2 ms, 77 K)	2.2 %
DBTDBT	Solution (In 2-MeTHF, 1×10^{-5} M)	349 nm (0.5 ns)<br>358 nm (0.5 ns, 77 K)<br>457 nm (265.3 ms, 77 K)<br>481 nm (255.0 ms, 77 K)	3.3 %
	Crystal	387 nm (0.3 ns)<br>385 nm (0.2 ns, 77 K)<br>599 nm (9.8 ms)<br>530 nm (239.1 ms, 77 K)	1.8 %
DBFDBFCZ	Solution (In 2-MeTHF, 1×10^{-5} M)	368 nm (4.0 ns)<br>364 nm (6.8 ns, 77 K)<br>446 nm (1450.7 ms, 77 K)<br>477 nm (1517.4 ms, 77 K)	20.6 %
	Crystal	386nm (1.3 ns)<br>393 nm (2.6 ns, 77 K)<br>554 nm (10.1 ms)<br>506 nm (607.8 ms, 77 K)	8.0 %
	Treated film	365 nm (4.9 ns)	10.4 %

	(In PVA, 1:1000)	491 nm (1171.7 ms)	
DBTDBFCZ	Solution (In 2-MeTHF, 1×10^{-5} M)	371 nm (3.4 ns)<br>364 nm (6.3 ns, 77 K)<br>453 nm (359.6 ms, 77 K)<br>480 nm (346.2 ms, 77 K)	17.0 %
	Crystal	393 nm (1.5 ns)<br>399 nm (3.4 ns, 77 K)<br>575 nm (3.4 ms)<br>525 nm (135.5 ms, 77 K)	5.5 %
	Treated film (In PVA, 1:1000)	366 nm (3.7 ns)<br>491 nm (206.9 ms)	14.3 %
DBFDBTCZ	Solution (In 2-MeTHF, 1×10^{-5} M)	374 nm (0.7 ns)<br>360 nm (2.4 ns, 77 K)<br>453 nm (351.7 ms, 77 K)<br>481 nm (341.8 ms, 77 K)	2.6 %
	Crystal	400 nm (0.5 ns)<br>408 nm (0.5 ns, 77 K)<br>530 nm (3.9 ms)<br>510 nm (189.6 ms, 77 K)	2.0 %
	Treated film (In PVA, 1:1000)	375 nm (2.2 ns)<br>491 nm (215.8 ms)	13.5 %
DBTDBTCZ	Solution (In 2-MeTHF, 1×10^{-5} M)	375nm (0.7 ns)<br>360 nm (1.1 ns, 77 K)<br>456 nm (249.5 ms, 77 K)<br>481 nm (238.8 ms, 77 K)	2.3 %
	Crystal	397 nm (0.4 ns)<br>395 nm (0.3 ns, 77 K)<br>558 nm (5.8 ms)<br>525 nm (217.4 ms, 77 K)	2.1 %

	Treated film (In PVA, 1:1000)	375 nm (2.1 ns) 491 nm (156.4 ms)	7.4 %

Table S4. ΔE_{S1-T1} of CZ, DBF, DBT, DBFDBF, DBFDBT, DBTDBT, DBFDBFCZ, DBFDBTCZ, DBTDBFCZ and DBTDBTCZ in 2-methyltetrahydrofuran (10^{-5} M) at 77 K.

Name	Cz	DBF	DBT	DBFDBF	DBFDBT
ΔE_{S1-T1} (eV)	0.65	1.15	0.88	0.91	0.87
Name	DBTDBT	DBFDBF CZ	DBTDBF CZ	DBFDBT CZ	DBTDBT CZ
ΔE_{S1-T1} (eV)	0.92	0.66	0.65	0.65	0.67

Table S5. Calculated SOC of molecules the optimized geometry of S_n and T_n at B3LYP/x2c-SVPall.

DBFDBFCZ					
(cm^{-1})	S_0	S_1	S_2	S_3	S_4
T_1	0.64	0.41	0.19	0.21	0.17
T_2	0.69	0.06	0.07	0.25	1.95
T_3	0.02	0.58	0.60	0.24	0.20
T_4	0.94	0.63	0.65	0.64	0.20
T_5	0.47	0.12	0.32	0.31	0.14
T_6	0.23	0.49	0.29	0.38	0.16

DBTDBFCZ					
(cm^{-1})	S_0	S_1	S_2	S_3	S_4
T_1	2.16	0.45	2.78	0.19	0.64
T_2	1.00	0.64	2.18	0.32	0.84
T_3	1.54	0.09	0.27	0.21	0.47
T_4	0.24	0.76	0.63	0.70	0.63

T_5	0.54	0.09	0.28	0.21	0.26
T_6	0.24	0.65	1.19	0.29	0.36

DBFDBTCZ					
(cm^{-1})	S_0	S_1	S_2	S_3	S_4
T_1	1.82	1.07	1.01	0.57	1.61
T_2	0.49	1.29	1.71	0.50	1.08
T_3	0.39	0.06	0.10	1.78	0.70
T_4	0.34	0.50	1.53	0.64	1.54
T_5	1.04	0.16	0.24	0.10	0.22
T_6	0.22	0.85	1.32	0.36	0.55

DBTDBTCZ					
(cm^{-1})	S_0	S_1	S_2	S_3	S_4
T_1	1.62	0.96	1.96	2.27	0.05
T_2	1.30	1.11	2.22	0.90	0.08
T_3	1.38	0.04	0.06	0.01	1.91
T_4	0.09	0.61	1.98	0.61	0.27
T_5	1.26	0.20	0.29	0.16	0.14
T_6	0.30	1.01	1.85	0.45	0.10

CZ					
(cm^{-1})	S_0	S_1	S_2	S_3	S_4
T_1	0.18	2.31	0.00	0.00	1.55
T_2	0.14	0.00	2.07	0.25	0.00
T_3	0.00	0.61	0.00	0.00	0.67
T_4	0.66	0.00	0.58	1.65	0.00
T_5	0.00	0.00	1.81	1.29	0.00
T_6	0.00	1.21	0.00	0.00	0.47

DBF					
(cm^{-1})	S_0	S_1	S_2	S_3	S_4
T_1	0.05	2.35	0.00	0.00	1.19
T_2	0.33	0.00	1.83	0.49	0.00
T_3	0.00	0.55	0.00	0.00	0.54
T_4	0.54	0.00	0.63	1.35	0.00
T_5	0.00	0.00	1.60	1.52	0.00
T_6	0.00	0.96	0.00	0.00	0.72

DBT					
(cm^{-1})	S_0	S_1	S_2	S_3	S_4
T_1	0.19	2.09	0.00	0.00	1.34
T_2	0.78	0.00	1.88	0.25	0.00
T_3	8.50	0.00	0.08	1.81	0.00
T_4	30.99	0.68	0.00	0.00	0.76
T_5	0.45	0.58	0.00	0.00	0.91
T_6	0.81	0.00	2.03	1.01	0.00

Table S6. Calculated photophysical parameters of doping treated PVA films at room temperature.

Sample	$k_p[s^{-1}]$	$k_{NR(T-S)}[s^{-1}]$	$k_{ISC}[s^{-1}]$
DBFDBFCZ	0.04	0.81	1.92×10^8
DBTDBFCZ	0.52	4.31	2.60×10^8
DBFDBTCZ	0.57	4.07	4.48×10^8
DBTDBTCZ	0.42	5.97	4.72×10^8

xyz coordinates

CZ

C	-3.04717	1.15565	-0.00004
C	-3.43174	-0.19855	0.00020
C	-2.48402	-1.22263	0.00024
C	-1.13325	-0.85986	0.00001
C	-0.72568	0.50547	-0.00018
C	-1.69931	1.51314	-0.00022
H	-3.81510	1.93226	0.00002
H	-4.49388	-0.45504	0.00037
H	-2.78886	-2.27164	0.00037
H	-1.40430	2.56519	-0.00023
C	1.13320	-0.85990	-0.00022
C	2.48399	-1.22266	0.00001
C	3.43174	-0.19861	0.00011
C	3.04724	1.15561	0.00012
C	1.69937	1.51312	0.00008
C	0.72574	0.50549	-0.00001
H	2.78881	-2.27169	0.00003
H	4.49385	-0.45518	0.00020
H	3.81516	1.93223	0.00031
H	1.40441	2.56518	0.00013
N	-0.00010	-1.65612	-0.00021
H	-0.00013	-2.66623	-0.00032

DBF

C	3.06176	-1.11056	-0.00014
C	3.40427	0.25390	-0.00013
C	2.42355	1.25042	-0.00008
C	1.09959	0.82655	-0.00004
C	0.72672	-0.53587	-0.00004
C	1.72640	-1.51718	-0.00009
H	3.85506	-1.86124	-0.00018
H	4.45784	0.54263	-0.00016
H	2.67434	2.31206	-0.00008
H	1.46597	-2.57790	-0.00009
C	-1.09959	0.82655	0.00004
C	-2.42355	1.25042	0.00011
C	-3.40427	0.25390	0.00016
C	-3.06176	-1.11056	0.00012
C	-1.72640	-1.51718	0.00006
C	-0.72672	-0.53587	0.00002
H	-2.67434	2.31206	0.00013

H	-4.45784	0.54263	0.00021
H	-3.85506	-1.86124	0.00015
H	-1.46597	-2.57790	0.00004
O	0.00000	1.64520	-0.00001

DBT

C	2.98779	-1.44400	-0.00013
C	3.49632	-0.13322	-0.00014
C	2.63734	0.96447	-0.00010
C	1.25750	0.73360	-0.00005
C	0.72729	-0.58008	-0.00003
C	1.61406	-1.66941	-0.00008
H	3.67724	-2.29110	-0.00016
H	4.57672	0.02885	-0.00019
H	3.03192	1.98260	-0.00011
H	1.22320	-2.68950	-0.00007
C	-1.25750	0.73360	0.00006
C	-2.63734	0.96447	0.00011
C	-3.49632	-0.13322	0.00014
C	-2.98779	-1.44400	0.00011
C	-1.61406	-1.66941	0.00006
C	-0.72729	-0.58008	0.00003
H	-3.03192	1.98260	0.00014
H	-4.57672	0.02885	0.00018
H	-3.67724	-2.29110	0.00014
H	-1.22321	-2.68950	0.00003
S	0.00000	1.96763	0.00001

DBFDBFCZ

C	-2.85966	1.03601	1.81329
C	-1.82605	1.82856	2.33404
C	-0.49714	1.61789	1.96800
C	-0.22270	0.58614	1.06325
C	-1.27886	-0.20289	0.55792
C	-2.62516	-0.01468	0.90475
C	0.98269	0.07091	0.43587
C	0.55065	-0.98452	-0.39416
O	-0.80611	-1.14881	-0.32094
C	2.34499	0.37915	0.50195
C	3.23646	-0.36157	-0.28260
C	2.77499	-1.40418	-1.11334
C	1.42107	-1.73895	-1.17194
C	-8.84234	2.01705	-0.44215
C	-9.33805	0.78299	-0.90107

C	-8.52427	-0.35057	-0.94014
C	-7.19601	-0.23579	-0.51030
C	-6.72662	1.01404	-0.05565
C	-7.51994	2.15417	-0.00899
C	-6.06199	-1.13818	-0.39615
C	-5.00559	-0.35576	0.11930
O	-5.41077	0.94070	0.32314
C	-5.84626	-2.49067	-0.68308
C	-4.57566	-3.01597	-0.44804
C	-3.53986	-2.21737	0.05779
C	-3.71688	-0.85361	0.36359
C	5.39232	3.47134	-1.20338
C	6.78952	3.39927	-1.04932
C	7.39940	2.21105	-0.64907
C	6.60210	1.08736	-0.39444
C	5.19065	1.18128	-0.54382
C	4.57465	2.36790	-0.95582
C	6.88985	-0.27531	0.01059
C	5.64099	-0.95163	0.09022
N	4.62094	-0.05962	-0.24840
C	8.06952	-0.96577	0.31841
C	7.99213	-2.30402	0.70209
C	6.74688	-2.95449	0.78718
C	5.55676	-2.29088	0.48594
H	-3.88487	1.23453	2.12197
H	-2.07450	2.62404	3.03977
H	0.30549	2.23855	2.37136
H	2.71931	1.17270	1.15009
H	3.49883	-1.94735	-1.72256
H	1.05556	-2.54436	-1.81011
H	-9.50253	2.88713	-0.42374
H	-10.37660	0.71375	-1.23183
H	-8.91440	-1.30594	-1.29775
H	-7.12102	3.10466	0.34789
H	-6.64959	-3.11403	-1.08095
H	-4.37649	-4.06880	-0.65922
H	-2.56118	-2.66432	0.22586
H	4.93539	4.40993	-1.52616
H	7.39949	4.28278	-1.24983
H	8.48475	2.15340	-0.53770
H	3.49319	2.42806	-1.08439
H	9.03575	-0.45900	0.26099
H	8.90432	-2.85393	0.94368
H	6.70806	-4.00122	1.09823

H	4.59415	-2.79819	0.56131

DBFDBTCZ

C	-2.69605	0.78539	1.97124
C	-1.55285	1.47479	2.39773
C	-0.30118	1.16080	1.87765
C	-0.19052	0.14577	0.91562
C	-1.35335	-0.54924	0.49957
C	-2.62611	-0.24452	1.02177
C	1.00058	-0.31184	0.21766
C	0.70713	-1.33323	-0.71717
S	-1.00124	-1.74967	-0.75033
C	2.32618	0.11807	0.36783
C	3.33160	-0.44626	-0.41889
C	3.02124	-1.45501	-1.35359
C	1.71419	-1.91002	-1.49880
C	-8.41174	2.59198	-0.66309
C	-9.11444	1.41007	-0.96128
C	-8.50617	0.15987	-0.83752
C	-7.17407	0.10351	-0.40760
C	-6.49415	1.30524	-0.11584
C	-7.08192	2.55929	-0.23270
C	-6.20980	-0.95507	-0.15647
C	-5.03277	-0.29872	0.26408
O	-5.20663	1.06181	0.28812
C	-6.22396	-2.35180	-0.25205
C	-5.06004	-3.04641	0.07790
C	-3.90162	-2.36917	0.48959
C	-3.84699	-0.96726	0.59201
C	5.06604	3.67990	-0.81564
C	6.45580	3.74450	-0.60276
C	7.18237	2.59266	-0.30394
C	6.50966	1.36718	-0.21025
C	5.10296	1.32200	-0.41549
C	4.37114	2.47291	-0.72759
C	6.93730	0.00742	0.05756
C	5.77251	-0.80709	0.00352
N	4.66890	0.00002	-0.28412
C	8.17707	-0.57845	0.34513
C	8.24092	-1.95162	0.57820
C	7.07645	-2.74087	0.53391
C	5.82917	-2.18322	0.24960
H	-3.66983	1.04809	2.38587
H	-1.65031	2.26584	3.14420

H	0.58788	1.70388	2.20534
H	2.58360	0.88598	1.09868
H	3.81983	-1.86968	-1.97039
H	1.48028	-2.69279	-2.22303
H	-8.91395	3.55610	-0.77027
H	-10.15228	1.47496	-1.29509
H	-9.05633	-0.75434	-1.07058
H	-6.52381	3.46698	0.00069
H	-7.12240	-2.88172	-0.57494
H	-5.04465	-4.13691	0.02206
H	-3.01537	-2.94704	0.75617
H	4.51683	4.59289	-1.05793
H	6.96784	4.70631	-0.67748
H	8.26263	2.64162	-0.14787
H	3.29548	2.42895	-0.90232
H	9.07978	0.03546	0.38918
H	9.20128	-2.42103	0.80231
H	7.14716	-3.81364	0.72900
H	4.92952	-2.79929	0.22601

DBTDBFCZ

C	2.60277	0.83189	-2.23378
C	1.54417	1.58309	-2.76874
C	0.23663	1.41444	-2.31386
C	0.00607	0.46781	-1.30832
C	1.08744	-0.27711	-0.79041
C	2.41132	-0.12365	-1.21956
C	-1.16315	0.01058	-0.57613
C	-0.68434	-0.97141	0.31733
O	0.66738	-1.14355	0.18865
C	-2.52809	0.31391	-0.59663
C	-3.37473	-0.35533	0.29436
C	-2.86684	-1.32409	1.18555
C	-1.51101	-1.65518	1.20113
C	8.90659	1.66815	1.18694
C	9.15044	0.31187	1.46473
C	8.18176	-0.65080	1.19370
C	6.95129	-0.26524	0.63764
C	6.72256	1.10464	0.36504
C	7.69292	2.07551	0.63546
C	5.80644	-1.08937	0.28169
C	4.74444	-0.32472	-0.26287
S	5.13140	1.39875	-0.32877
C	5.64656	-2.47455	0.43617

C	4.44595	-3.07154	0.06382
C	3.40093	-2.30228	-0.46486
C	3.52543	-0.91735	-0.64800
C	-5.48611	3.54830	0.99379
C	-6.88950	3.46619	0.92425
C	-7.51748	2.24897	0.66350
C	-6.73257	1.10560	0.46398
C	-5.31528	1.20906	0.52591
C	-4.68054	2.42569	0.79829
C	-7.03886	-0.28657	0.19637
C	-5.79470	-0.97024	0.10672
N	-4.75961	-0.05429	0.30862
C	-8.23215	-0.99857	0.01697
C	-8.17300	-2.36551	-0.25142
C	-6.93282	-3.02389	-0.34877
C	-5.72940	-2.33925	-0.17413
H	3.61010	0.98065	-2.62567
H	1.75468	2.30743	-3.55831
H	-0.58444	2.00217	-2.72928
H	-2.93833	1.04966	-1.28964
H	-3.55596	-1.81162	1.87661
H	-1.10977	-2.40363	1.88548
H	9.67531	2.41311	1.40503
H	10.10765	0.01192	1.89671
H	8.37619	-1.70336	1.41168
H	7.50310	3.12903	0.41997
H	6.45728	-3.07483	0.85460
H	4.31213	-4.14869	0.18401
H	2.46512	-2.78554	-0.74784
H	-5.01447	4.51038	1.20788
H	-7.48974	4.36514	1.08053
H	-8.60718	2.18420	0.61806
H	-3.59380	2.49452	0.86119
H	-9.19492	-0.48641	0.08384
H	-9.09589	-2.93234	-0.39215
H	-6.90884	-4.09398	-0.56840
H	-4.77132	-2.85365	-0.25856

DBTDBTCZ

C	-2.44750	0.62165	2.28533
C	-1.28553	1.27876	2.71685
C	-0.05780	1.01306	2.11836
C	0.01313	0.07776	1.07442
C	-1.16794	-0.58115	0.65362

C	-2.41591	-0.32060	1.24847
C	1.17122	-0.33168	0.29588
C	0.83216	-1.28393	-0.69588
S	-0.87987	-1.69012	-0.69014
C	2.50472	0.08145	0.42136
C	3.47337	-0.42984	-0.44371
C	3.11797	-1.36911	-1.43333
C	1.80317	-1.80777	-1.55653
C	-8.64224	2.17243	-1.18503
C	-9.09267	0.84892	-1.33257
C	-8.26903	-0.22038	-0.99129
C	-6.97818	0.02387	-0.49526
C	-6.54028	1.36314	-0.35376
C	-7.36509	2.44044	-0.69515
C	-5.95800	-0.92755	-0.08281
C	-4.77954	-0.28213	0.36625
S	-4.90089	1.47680	0.28029
C	-6.00561	-2.33018	-0.09851
C	-4.89742	-3.05983	0.32128
C	-3.73590	-2.40594	0.75815
C	-3.65144	-1.00742	0.79134
C	5.21899	3.70722	-0.61688
C	6.61671	3.75070	-0.45754
C	7.34672	2.57747	-0.27170
C	6.66972	1.35124	-0.23770
C	5.25558	1.32663	-0.38754
C	4.51976	2.49988	-0.58637
C	7.09840	-0.02594	-0.08517
C	5.92679	-0.82981	-0.14916
N	4.81801	0.00035	-0.33321
C	8.34471	-0.63578	0.10923
C	8.40839	-2.02232	0.24154
C	7.23764	-2.80167	0.18905
C	5.98377	-2.22014	-0.00379
H	-3.40185	0.83720	2.76944
H	-1.34946	2.00328	3.53133
H	0.84435	1.53027	2.45223
H	2.79656	0.79500	1.19325
H	3.88793	-1.74249	-2.11002
H	1.53505	-2.53658	-2.32392
H	-9.29889	3.00205	-1.45704
H	-10.09674	0.65917	-1.71813
H	-8.62350	-1.24705	-1.10776
H	-7.01529	3.46854	-0.58106

H	-6.90652	-2.84237	-0.44315
H	-4.92871	-4.15139	0.31426
H	-2.87768	-2.99256	1.09134
H	4.66675	4.63741	-0.77067
H	7.13205	4.71326	-0.48475
H	8.43277	2.61063	-0.15730
H	3.43756	2.47308	-0.71931
H	9.25269	-0.03021	0.15977
H	9.37375	-2.51037	0.39232
H	7.30868	-3.88588	0.30454
H	5.07969	-2.82941	-0.03413

Supplementary equation

As the importance of the k_{ISC} value discussed in the manuscript, details, and calculation equations are expressed here. As we known,

$$\Phi_{ISC} = \frac{k_{ISC}}{k_{ISC} + k_F + k_{IC}}$$

$$\Phi_F = \frac{k_F}{k_{ISC} + k_F + k_{IC}}$$

considering the larger proportion of phosphorescence emission relative to fluorescence, we assume that

$\Phi_{ISC} + \Phi_F = 1.$

Therefore,

$$\Phi_{ISC} = \frac{k_{ISC}}{k_{ISC} + k_F} = k_{ISC} \cdot \tau_F$$

$$\Phi_F = \frac{k_F}{k_{ISC} + k_F}$$

After conversion,

$$k_{ISC} = \frac{1 - \Phi_F}{\tau_F}$$

Because

$$\Phi_p = \Phi_{ISC}\frac{k_p}{k_p + k_{nr}}$$

$$k_p = \frac{1 - k_{nr}\tau_p}{\tau_p}$$

Therefore,

$$k_p = \frac{\Phi_p}{(1 - \Phi_F)\tau_p}$$

$$k_{nr} = \frac{1}{\tau_p} - \frac{\Phi_p}{(1 - \Phi_F)\tau_p}$$

References

1. R. Kabe and C. Adachi, *Nature*, 2017, **550**, 384-387.
2. P. Alam, T. S. Cheung, N. L. C. Leung, J. Zhang, J. Guo, L. Du, R. T. K. Kwok, J. W. Y. Lam, Z. Zeng, D. L. Phillips, H. H. Y. Sung, I. D. Williams and B. Z. Tang, *J. Am. Chem. Soc.*, 2022, **144**, 3050-3062.
3. Z. Lin, R. Kabe, N. Nishimura, K. Jinnai and C. Adachi, *Adv. Mater.*, 2018, **30**, 1803713.
4. P. Alam, N. L. C. Leung, J. Liu, T. S. Cheung, X. Zhang, Z. He, R. T. K. Kwok, J. W. Y. Lam, H. H. Y. Sung, I. D. Williams, C. C. S. Chan, K. S. Wong, Q. Peng and B. Z. Tang, *Adv. Mater.*, 2020, **32**, 2001026.
5. L. Ma, Q. Xu, S. Sun, B. Ding, Z. Huang, X. Ma and H. Tian, *Angew. Chem. Int. Ed.*, 2022, **61**, e202115748.
6. C. Qian, Z. Ma, X. Fu, X. Zhang, Z. Li, H. Jin, M. Chen, H. Jiang, X. Jia and Z. Ma, *Adv. Mater.*, 2022, **34**, 2200544.
7. Z. Xie, Z. Mao, H. Wang, Y. Xiao, X. Zhang, T. Yu, Z. An and W. Huang, *Light: Sci. Appl.*, 2024, **13**, 85.
8. C. Qian, X. Zhang, Z. Ma, X. Fu, Z. Li, H. Jin, M. Chen, H. Jiang and Z. Ma, *CCS Chem.*, 2023, **6**, 798-811.
